# A *Drosophila* model to study retinitis pigmentosa pathology associated with mutations in the core splicing factor Prp8

**DOI:** 10.1242/dmm.043174

**Published:** 2020-06-26

**Authors:** Dimitrije Stanković, Ann-Katrin Claudius, Thomas Schertel, Tina Bresser, Mirka Uhlirova

**Affiliations:** 1 Institute for Genetics and Cologne Excellence Cluster on Cellular Stress Responses in Aging-Associated Diseases (CECAD), University of Cologne, Cologne 50931, Germany; 2 Center for Molecular Medicine Cologne, University of Cologne, Cologne 50931, Germany

**Keywords:** *Drosophila*, Prp8, Retinitis pigmentosa, Apoptosis, Eye development, Splicing factor

## Abstract

Retinitis pigmentosa (RP) represents genetically heterogeneous and clinically variable disease characterized by progressive degeneration of photoreceptors resulting in a gradual loss of vision. The autosomal dominant RP type 13 (RP13) has been linked to the malfunction of PRPF8, an essential component of the spliceosome. Over 20 different RP-associated *PRPF8* mutations have been identified in human patients. However, the cellular and molecular consequences of their expression *in vivo* in specific tissue contexts remain largely unknown. Here, we establish a *Drosophila melanogaster* model for RP13 by introducing the nine distinct RP mutations into the fly *PRPF8* ortholog *prp8* and express the mutant proteins in precise spatiotemporal patterns using the Gal4/UAS system. We show that all nine RP-Prp8 mutant proteins negatively impact developmental timing, albeit to a different extent, when expressed in the endocrine cells producing the primary insect moulting hormone. In the developing eye primordium, uncommitted epithelial precursors rather than differentiated photoreceptors appeared sensitive to Prp8 malfunction. Expression of the two most pathogenic variants, Prp8^S>F^ and Prp8^H>R^, induced apoptosis causing alterations to the adult eye morphology. The affected tissue mounted stress and cytoprotective responses, while genetic programs underlying neuronal function were attenuated. Importantly, the penetrance and expressivity increased under *prp8* heterozygosity. In contrast, blocking apoptosis alleviated cell loss but not the redox imbalance. Remarkably, the pathogenicity of the RP-Prp8 mutations in *Drosophila* correlates with the severity of clinical phenotypes in patients carrying the equivalent mutations, highlighting the suitability of the *Drosophila* model for in-depth functional studies of the mechanisms underlying RP13 etiology.

This article has an associated First Person interview with the first author of the paper.

## Abstract (precis)

**Summary:** A *Drosophila melanogaster* disease model of retinitis pigmentosa type 13 to study the mechanisms underlying the loss of tissue homeostasis and degeneration caused by mutations in the core component of the spliceosome Prp8.

## INTRODUCTION

Retinitis pigmentosa (RP; OMIM 268000) represents a heterogeneous group of hereditary eye disorders characterized by a progressive degeneration of the light-sensing photoreceptor cells in the retina. Early RP symptoms include night blindness and gradual loss of peripheral vision due to the loss of rod photoreceptors which ensure achromatic, low-light vision. As the disease advances, the rod elimination is followed by the death of cones, ultimately resulting in complete blindness (Campochiaro and Mir, 2018; Hartong et al., 2006). The rate and extent of disease progression vary markedly among RP patients. Mutations in 30 different genes have been linked to the autosomal dominant form of RP (adRP) (RetNet: https://sph.uth.edu/retnet/home.htm). Intriguingly, nearly one-quarter of these genes encode core components of the spliceosome, a macromolecular RNA-protein complex that removes introns from nascent pre-mRNAs, generating mature transcripts. The spliceosome consists of five small nuclear ribonucleoprotein particles (snRNPs) – U1, U2, U4, U5 and U6, – and more than 100 associated proteins (Wahl et al., 2009; Will and Lührmann, 2011). Each of the five major snRNPs is built up from a single uridine-rich small nuclear RNA (U-snRNA) and a specific set of proteins. During the splicing reaction, the spliceosome assembles in a stepwise fashion on each intron. Initially, the U1 and U2 snRNPs recognize and bind the 5′ splice site (5′SS) and the branch point of the pre-mRNA, respectively. The subsequent recruitment of the pre-assembled U4/U6.U5 tri-snRNP triggers changes in conformation and composition of snRNAs and snRNPs converting the pre-spliceosome to a catalytically active complex that executes the splicing reaction (Matera and Wang, 2014; Will and Lührmann, 2011). Remarkably, all of the pre-mRNA splicing-associated genes mutated in adRP are components of the U4/U6.U5 tri-snRNP including *PRPF3*, *PRPF4*, *PRPF6*, *PRPF8*, *PRPF31*, retinitis pigmentosa 9 protein (*PAP-1*; also known as *RP9*) and the U5 small nuclear ribonucleoprotein 200 kDa DEAD-box RNA helicase (*SNRNP200* or *Brr2*) (Růžič ková and Staněk, 2017). How malfunctions in core splicing factors manifest in tissue-specific pathogenesis rather than a systemic disease remains puzzling.

Pre-mRNA processing factor 8 (PRPF8/Prp8) is the largest and the most conserved protein of the spliceosome involved in nearly all functions of the U5 snRNP. These include the splice site and branch region recognition, assembly and stabilization of the U4/U6.U5 tri-snRNP, exon alignment and activation of the catalytic core of the spliceosome. Prp8 does so through precisely controlled interactions with the U5 snRNA and several proteins of the splicing machinery including Brr2, and a GTPase EFTUD2 (Snu114) (Boon et al., 2006; Grainger and Beggs, 2005).

Twenty-two distinct *PRPF8* mutations have been identified so far in patients suffering from RP13. The majority of these mutations map to the terminal exon 43 encoding the Jab1/MPN domain (Escher et al., 2018; Růžič ková and Staněk, 2017; Van Cauwenbergh et al., 2017). Studies in yeast, cultured mammalian cells and biochemical approaches have yielded fundamental mechanistic insights into the properties of wild-type and mutant RP-Prp8 proteins. It has been demonstrated that some of the RP-Prp8 mutations alter interactions of the Jab1/MPN domain with Snu114 and Brr2, causing defects in snRNP assembly or premature spliceosome activation, ultimately resulting in reduced splicing efficiency or splicing defects (Malinová et al., 2017; Mayerle and Guthrie, 2016; Mozaffari-Jovin et al., 2013). However, not all RP-Prp8 mutations significantly perturbed the known Prp8 protein interactome, indicating that diverse mechanisms might underpin the pathogenicity of the different mutant variants. The cellular and molecular consequences of different RP-Prp8 mutations *in vivo* within a specific tissue context has not been systematically addressed.

The fruit fly *Drosophila melanogaster* has proven itself as the organism of choice for modelling and unravelling the underlying causes of complex human diseases as diverse as cancer or neurodegeneration (Bilen and Bonini, 2005; Gaspar et al., 2019; Gonzalez, 2013; Rudrapatna et al., 2012). Owing to the sophisticated genetic tools available, their fast generation time and the remarkable functional conservation of genes and signalling pathways, the fly model facilitates rapid screening and functional characterization of human disease-related genes in defined genetic, developmental and tissue contexts (Yamamoto et al., 2014). Importantly, genetic studies in *Drosophila* have helped to uncover function of several genes whose mutations cause dominant or recessive forms of RP, including *crumbs* (*crb*) (*RP12*), *rhodopsin* (*rh*/*RHO*) (*RP4*) and *eyes shut* (*eys*) (*RP25*) (Gaspar et al., 2019; Lehmann et al., 2019). The fly model is, therefore, perfectly suited for a rapid assessment of tissue-specific pathogenicity of RP mutations and mechanisms by which they affect cell and tissue homeostasis.

Here, we establish a *Drosophila melanogaster* model for RP13. We demonstrate that nine different RP-associated Prp8 mutant proteins delay the developmental transition when expressed in the endocrine organ specialized to produce the major insect moulting hormone. In the developing eye primordium, actively cycling cells rather than differentiated photoreceptors showed sensitivity to Prp8 malfunction. The overexpression of the two most toxic RP-Prp8 variants induced apoptosis and disturbances of the adult eye morphology. Whereas the affected tissue mounted the stress and cytoprotective response, the genetic programs underlying neuronal function were attenuated. Importantly, the expressivity and penetrance among the RP-Prp8 mutations differed and increased under *prp8* heterozygosity.

## RESULTS

### *Drosophila* toolbox to elucidate phenotypic consequences of RP-associated Prp8 mutations

**Fig. 1.**
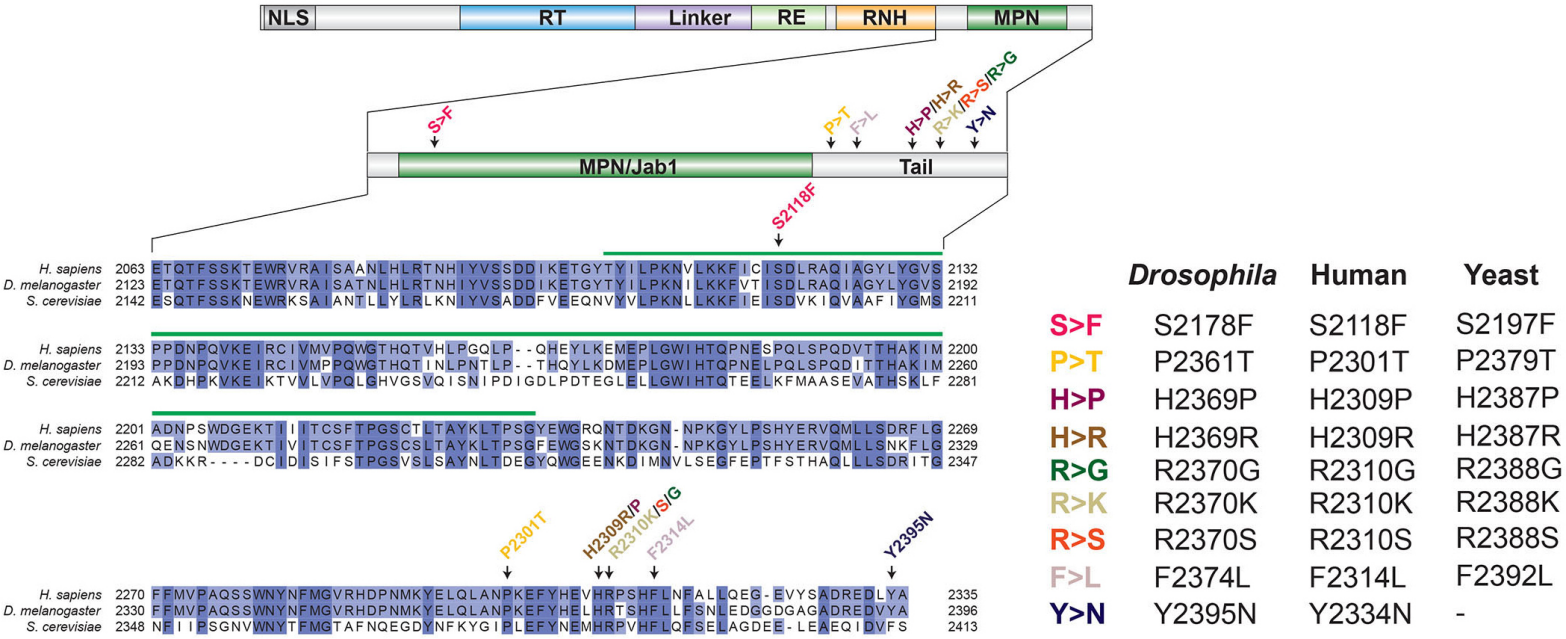
**The *Drosophila* model for an autosomal dominant retinitis pigmentosa type 13.** The scheme of the Prp8 protein and its domains. Alignment of the C-terminal part of the human (Q6P2Q9), *Drosophila* (A1Z8U0) and yeast (P33334) Prp8 orthologs indicating positions of the nine RP-associated mutations affecting the conserved amino acids (left). Individual RP mutations received a unique name according to the mutated amino acid (right).

The *Drosophila* Prp8 protein comprises 2396 amino acids and shares 88.99% and 59.50% identity with its human and yeast counterpart, respectively (Fig. 1). To mimic nine different human PRPF8 RP-associated mutations (S2118F, P2301T, F2314L, H2309P, H2309R, H2310G, H2310K, R2310S, Y2334N), we used site-directed mutagenesis to introduce the corresponding missense substitutions into the *Drosophila prp8* coding sequence (S2178F, P2361T, F2374L, H2369P, H2369R, H2370G, H2370K, R2370S, Y2395N) (Fig. 1). Each mutant has been assigned a unique name according to the mutated amino acid (e.g. S2178F is hereafter referred to as Prp8^S>F^), to simplify the description (Fig. 1). To study how distinct RP-Prp8 mutations affect different tissues *in vivo* and whether they share common pathomechanisms, we exploited the Gal4/UAS system (Brand and Perrimon, 1993), which allows targeted expression of the transgenes in spatially and temporally defined manner. To this end, wild-type and mutant *prp8* cDNAs were cloned into the *pUAST-attB* vector (Bischof et al., 2007) and integrated into the same *attP-9A* landing site (Venken et al., 2006) to ensure uniform inducible expression. We also generated the UAS-based transgenic constructs allowing expression of the wild-type and seven of the RP-Prp8 mutant variants with N-terminal Flag-tag, which were integrated into the *attP2* landing site (Groth et al., 2004). We selected three *Gal4* driver lines, namely *phantom (phm)-Gal4*, *eyeless* (*ey*)-*Gal4* and *Glass multiple reporter* (*GMR*)-*Gal4* to overexpress the Prp8 transgenes in specific cells during the fly development*.* While *phm-Gal4* expresses in the endoreplicating polyploid cells of the prothoracic gland (PG) (Fig. S1A) *ey-Gal4* and *GMR-Gal4* are active in the eye/antennal imaginal discs (EADs) which will give rise to the adult compound eyes, antennae and epithelia of the head capsule. *Ey-Gal4* becomes active during embryogenesis in all cycling cells of the eye/antennal primordium while showing a restricted eye-specific expression in the late third instar larva (Huang et al., 2017 and Fig. S1B). In contrast, *GMR-Gal4* is a late-acting driver which targets the expression to the EAD domain posterior of the morphogenetic furrow comprised predominantly of differentiating cells arrested in G1 or G2 phase of the cell cycle, and cells within the second mitotic wave, which undergo one round of cell division (Fig. S1C). The *Gal4* drivers were selected based on our previous study where we demonstrated differential response and sensitivity of the targeted cells to spliceosome deficiency (Claudius et al., 2014). Moreover, the targeted tissues are easily accessible to cell biology and molecular approaches and facilitate scoring of various phenotypic traits.

Taken together, we generated nine different RP-Prp8 mutant variants, the expression of which can be easily controlled in a spatiotemporal manner. Such an approach enables rapid and parallel investigation of their impact *in vivo*, on the developing retina but also other tissues that fulfil highly specialized functions.

### Prp8 predominantly resides in the cytoplasm of *Drosophila* cells

**Fig. 2.**
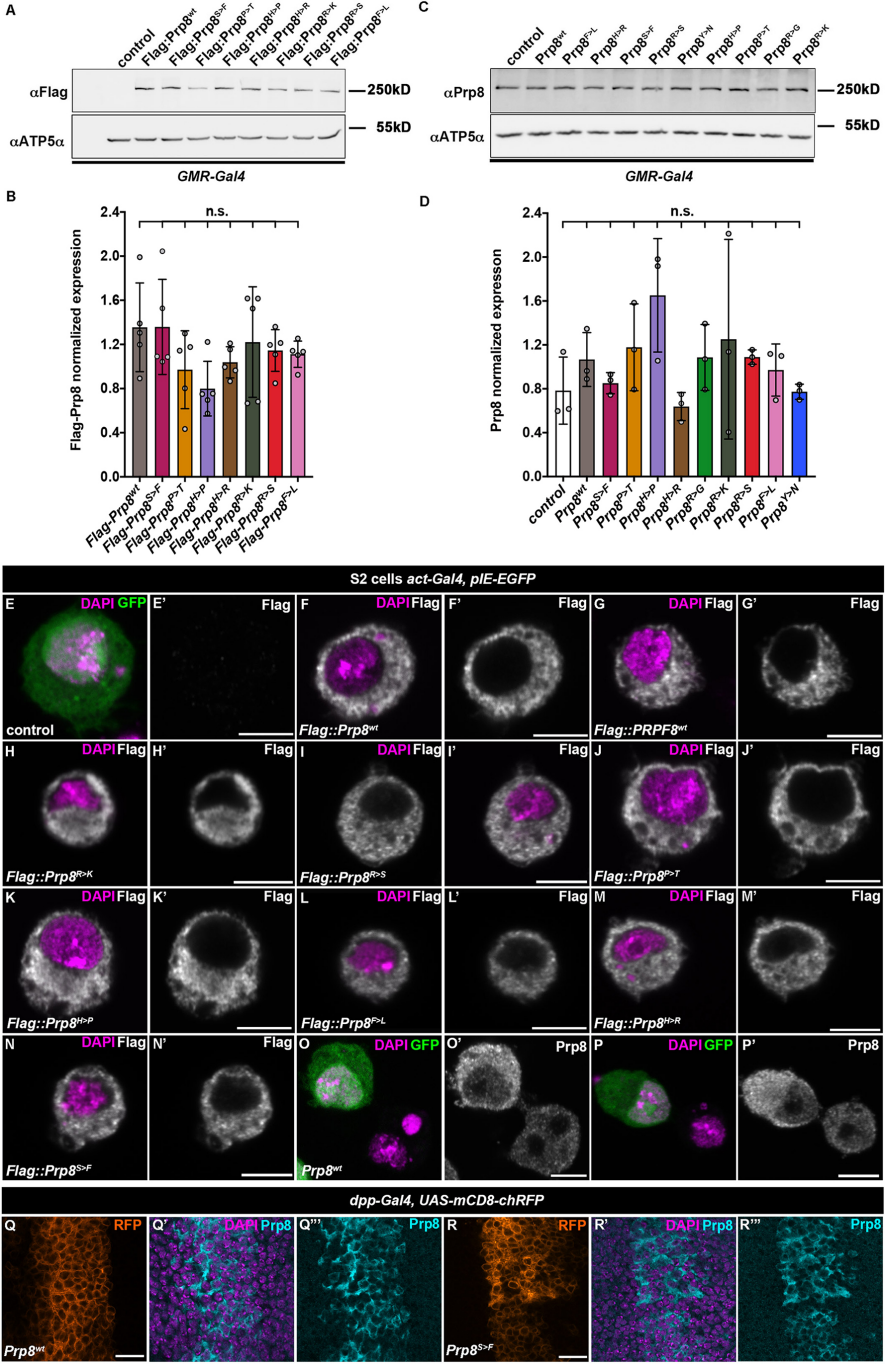
**Prp8 primarily localizes to the cytoplasm in *Drosophila* cells.** (A–D) Immunoblots showing comparable induction of Flag-tagged (A,B) and non-tagged (C,D) wild-type and RP-Prp8 variants in lysates prepared from the third instar larval EADs expressing the respective transgenes under the control of the *GMR-Gal4* driver. The transgenic Prp8 proteins do not markedly elevate the total Prp8 levels (C,D). ATP5α served as loading controls. Data represent means±s.d. of normalized Prp8 protein expression, *n*=5 (B), *n*=3 (D). Statistical significance was determined using ordinary one-way ANOVA with Tukey's multiple comparisons test, n.s., non-significant. (E–P) Transfected Flag-tagged human PRPF8^wt^ (G), *Drosophila* Prp8^wt^ (F) and RP-Prp8 mutant proteins (H–N) showing cytoplasmic localization in *Drosophila* S2 cells (GFP) similar to the non-tagged *Drosophila* Prp8^wt^ (O) or endogenous Prp8 (P) as determined by immunostaining with an anti-Flag (E–N) or Prp8-specific antibodies (O,P). Expression of UAS-based Prp8 transgenes was driven by actin promoter from co-transfected pAW-Gal4 plasmid while GFP-expressing pIE-GFP vector served to identify transfected cells. Nuclei were stained with DAPI. Scale bars: 5 µm. (Q–R) Overexpressed non-tagged Prp8^wt^ (Q) and Prp8^S>F^ (R) transgenic proteins using *dpp-Gal4* driver are enriched in cytoplasm of larval wing imaginal disc cells (Q‴, R‴). The *dpp* expression domain is marked by membrane-tethered RFP (Q,R); nuclei are stained with DAPI. Scale bars: 10 µm.

To assess the levels and localization of transgenic proteins, we performed western blot analysis of lysates prepared from third instar EADs expressing the Prp8 transgenes under the control of the *GMR*-*Gal4* driver. Immunoblotting with anti-Flag and self-generated anti-*Drosophila* Prp8-specific antibodies (Fig. S2A) confirmed that the wild-type and RP-Prp8 proteins were stably expressed and inducible to the same level irrespective of the tag or the type of mutation (Fig. 2A–D). Interestingly, despite being expressed from the strong *GMR-Gal4* driver, the transgenic proteins did not dramatically surpass the endogenous Prp8 levels (Fig. 2C,D). Immunostaining of *Drosophila* S2 cells (Fig. 2E–P) and imaginal discs (Fig. 2Q,R, Fig. S2B) further revealed that the transgenic wild-type Prp8 (Prp8^wt^) as well as the RP-Prp8 mutant variants primarily localized to the cytoplasm where the endogenous *Drosophila* Prp8 or transgenic human Flag::PRPF8 proteins were also mostly confined. Thus, in contrast to the nuclear enrichment in mammalian cells (Malinová et al., 2017), wild-type Prp8 protein as well as RP-Prp8 mutant variants are mainly cytoplasmic in *Drosophila* cells. As the overexpression of the transgenic Prp8 variants did not markedly increase the overall protein abundance, we suggest that the experimental strategy is suitable to uncover phenotypic consequences of RP-Prp8 variants with limited possibility of artefacts caused by a strong overexpression.

### RP-Prp8 mutations impact function of *Drosophila* prothoracic gland

**Fig. 3.**
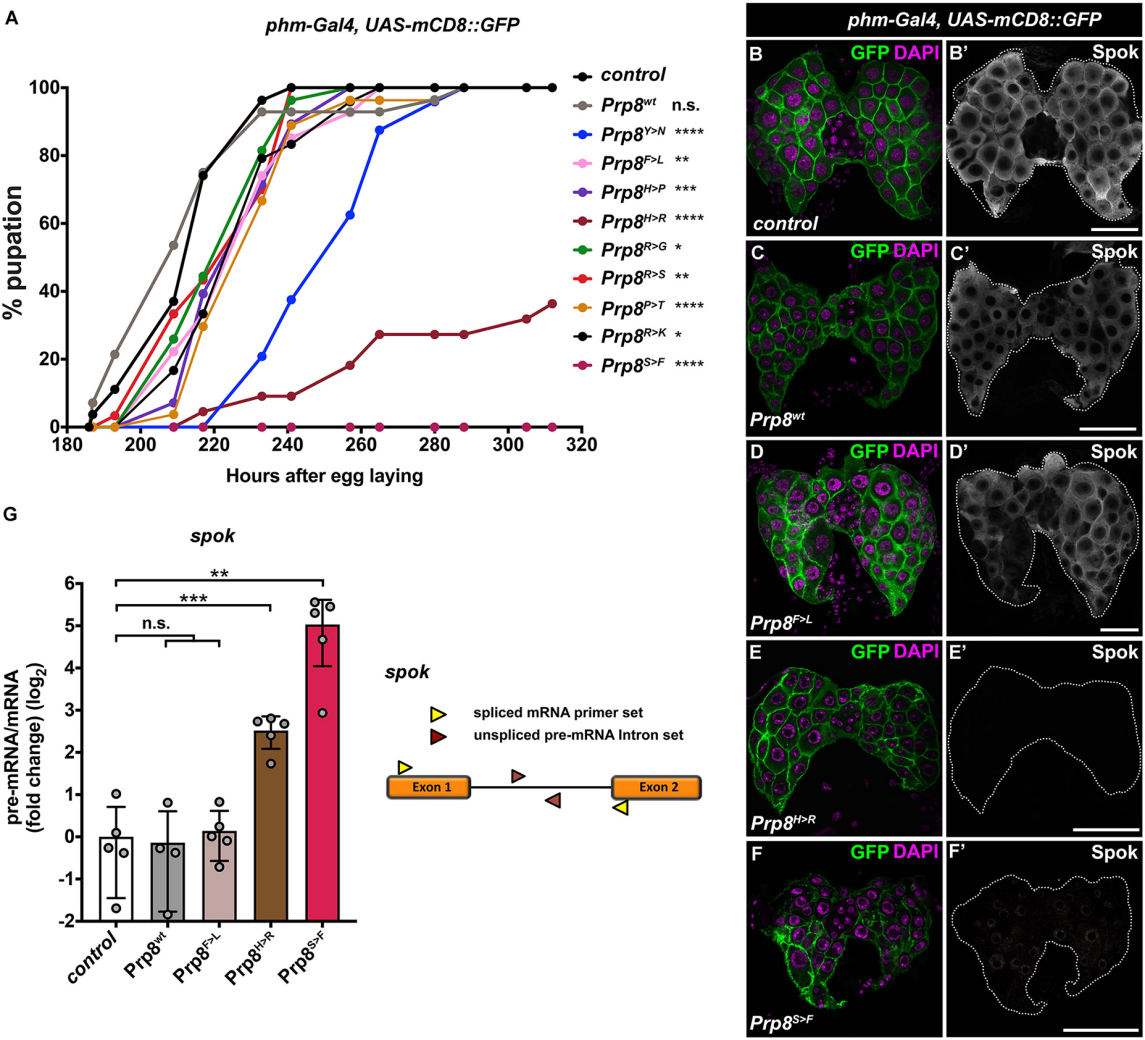
**Differential impact of RP-Prp8 mutations on the function of *Drosophila* prothoracic gland.** (A) All nine RP-Prp8 mutant variants, but not Prp8^wt^, delay pupation when overexpressed in the prothoracic gland (PG) using the *phm-Gal4* driver. Pupation rates are presented as the percentage of larvae (*n*≥22 per genotype) that form pupae over time. The pupae were counted at set intervals AEL. Pupation curves represent one of two independent experiments. Statistical significance was determined by Log-rank test. (B–F) Alteration of Spok protein levels in *Drosophila* PGs (7 days AEL) overexpressing non-tagged RP-Prp8 variants under the control of the *phm-Gal4* driver. Relative to control (B′), Prp8^wt^ (C′) and Prp8^F>L^ (D′), the Spok signal was barely detectable in PG glands expressing Prp8^H>R^ (E′) and Prp8^S>F^ (F′). Note the altered morphology of the PG and their nuclei following overexpression of Prp8^H>R^ (E) and Prp8^S>F^ (F). PG cells are highlighted with mCD8::GFP; DAPI stains the nuclei. Panels show projections of multiple confocal sections. Scale bars: 20 µm. (G) PG-specific expression of Prp8^H>R^ and Prp8^S>F^ causes accumulation of unspliced, intron-retaining *spok* transcript. The pre-mRNA:mRNA ratios shown as a log_2_ fold-change compared with the control were calculated from the normalized RT-qPCR data by dividing values obtained with intron primer set (red triangles) with values obtained using primers in adjacent exons (yellow triangles). Data are means±s.d., *n*=4–5. Statistical significance was determined using unpaired *t*-tests with Welch's correction assuming unequal variance. *P≤0.05, **P≤0.01, ***P<0.001, ****P<0.0001, n.s., non-significant in A and G. The exact number of animals per genotype (A) and biological replicates (G) per sample (*n*) and *P*-values are specified in Supplementary Dataset 2.

The prothoracic gland of *Drosophila* larvae, like the human retina, represents a highly specialized organ with a great demand for tissue-specific protein synthesis. The PG produces the steroid hormone ecdysone, which orchestrates major developmental transitions, including moulting and metamorphosis. We have shown previously that the PG cells are highly sensitive to Prp8 downregulation (*phm>prp8^RNAi^*), which causes missplicing of genes encoding key steroidogenic enzymes (Claudius et al., 2014). The developmental delay or arrest as a consequence of ecdysone deficiency has emerged as a readily scorable phenotype to investigate spliceosome activity. Strikingly, the PG-specific overexpression of all RP-Prp8 mutant variants resulted in developmental delay compared to control and the expression of the Prp8^wt^ protein. The phenotype was the strongest for Prp8^H>R^ and Prp8^S>F^, which significantly hindered or completely blocked pupation and subsequent adult eclosion (Fig. 3A). The altered timing of developmental transitions strongly correlated with barely detectable levels of the key steroidogenic enzyme Spookier (Spok) in Prp8^H>R^ and Prp8^S>F^ glands (Fig. 3E,F) relative to Prp8^F>L^ and Prp8^wt^-expressing PG cells (Fig. 3C,D), which had a comparable signal to the control (Fig. 3B). Importantly, in Prp8^H>R^- and Prp8^S>F^-expressing PGs we observed faulty processing of *spok* pre-mRNA (Fig. 3G), which may explain the absence of Spok protein in these genotypes.

In conclusion, the targeted expression of the nine RP-Prp8 variants in PG cells uncovered their differential negative impact on PG function and, consequently, on animal development. The Prp8^H>R^ and Prp8^S>F^ variants emerged as the most toxic, causing the deterioration of the overall organ morphology and abnormal processing of the *spok* transcript, thus mimicking the effect of *prp8* deficiency (Claudius et al., 2014).

### Early expression of Prp8^H>R^ and Prp8^S>F^ RP mutant variants induces apoptosis and defects in adult eye morphogenesis

**Fig. 4.**
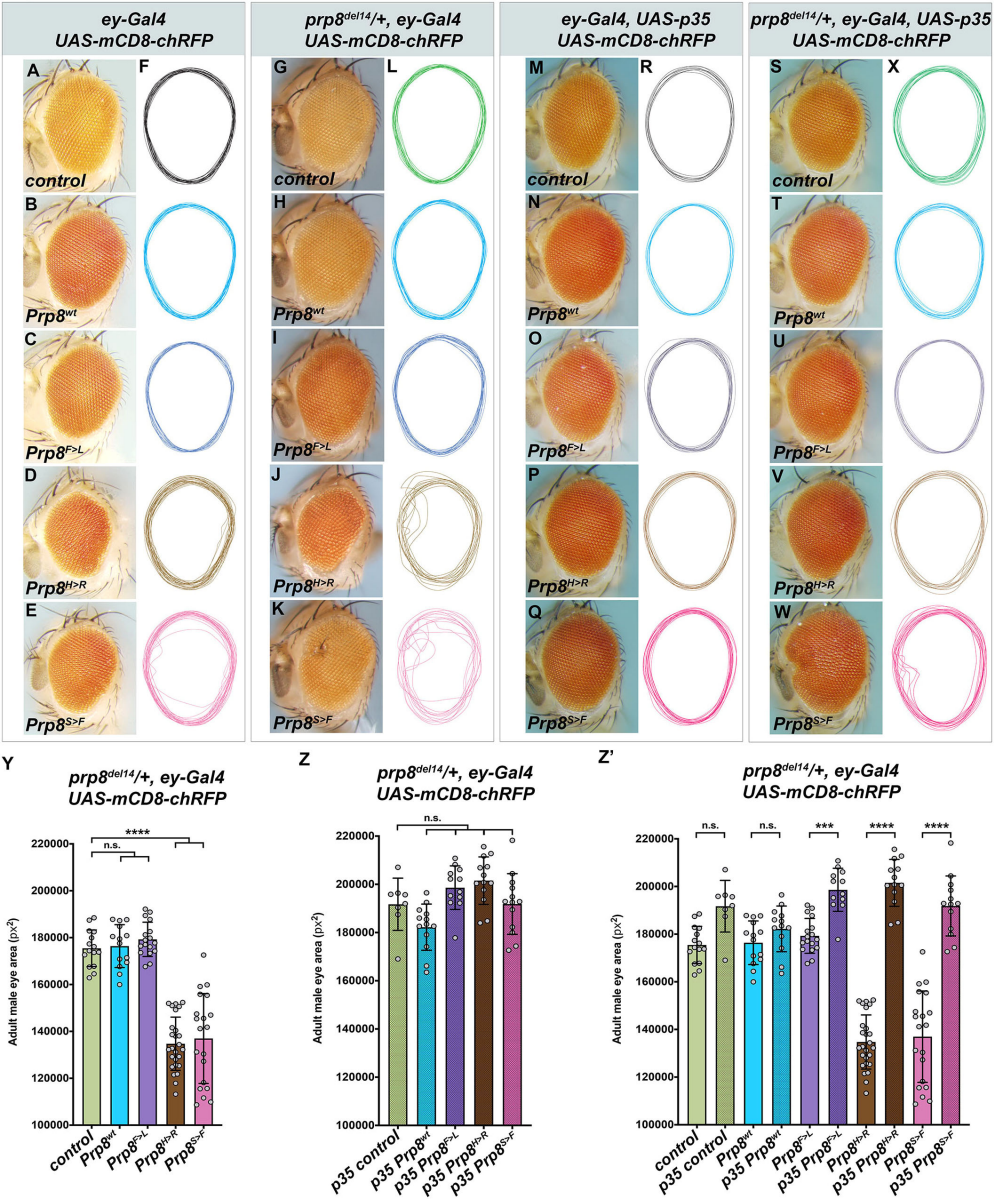
**Early induction of Prp8^S>F^ and Prp8^H>R^ expression causes adult eye defects.** (A–F) Expression of Prp8^H>R^ (D) and Prp8^S>F^ (E) in the EADs using the early acting *ey-Gal4* driver resulted in rough irregularly shaped adult eyes compared with control (A) and those expressing Prp8^wt^ (B) and Prp8^F>L^ RP variant (C). Outlines of adult eyes from the indicated genotypes highlight organ irregularities caused by *ey-*specific Prp8^H>R^ and Prp8^S>F^ expression (F). (G–L) The expressivity of the Prp8^H>R^- and Prp8^S>F^-induced phenotypes was markedly enhanced when overexpressed by the *ey-Gal4* driver in *prp8^del14^*/+ heterozygous flies carrying only one functional copy of endogenous *prp8* gene. (M–X) Blocking apoptosis by expressing the pan-caspase inhibitor p35 restored adult eye size and mitigated the morphological defects caused by *ey-*specific overexpression of Prp8^S>F^ and Prp8^H>R^ in wild-type (M-R) or *prp8^del14^*/+ heterozygous background (S–X). Outlines of adult eyes from the indicated genotypes are presented vertically aligned along their midline (F,L,R,X). (Y–Z′) *ey-*specific overexpression of Prp8^H>R^ and Prp8^S>F^ but not Prp8^wt^ or Prp8^F>L^ in *prp8^del14^*/+ heterozygous background lead to smaller adult eyes relative to control (Y). The ommatidia loss could be prevented and eye size normalized by co-expression of p35 (Z, Z′). Data represent means±s.d., *n*≥8. Statistical significance was determined using ordinary one-way ANOVA with Tukey's multiple comparisons test; ***P<0.001, ****P<0.0001, n.s., non-significant. The exact number of adult eyes per genotype (*n*) and *P*-values are specified in Supplementary Dataset 2.

To determine whether and how the RP-Prp8 mutations affect the development and differentiation of the adult compound eye, we overexpressed the wild-type and mutant variants under the control of the *ey-Gal4* and *GMR-Gal4* drivers. Interestingly, expression of any single RP-Prp8 mutant protein or *prp8^RNAi^* using a late-acting *GMR-Gal4* was asymptomatic. The adult flies eclosed and their eyes were indistinguishable from the control and those expressing Prp8^wt^ (Fig. S3A–L)*.* In contrast, early expression of Prp8^H>R^ and Prp8^S>F^ using *ey-Gal4* resulted in rough and irregularly shaped adult compound eyes (Fig. 4D,E, Fig. S4C,I). The Prp8^H>R^ and Prp8^S>F^ phenotypes sharply contrasted with undisturbed morphology of control adult eyes and those overexpressing Prp8^wt^ and seven other RP-Prp8 mutant variants (Fig. 4A–F, Fig. S4A–K). Of note, the *ey-Gal4*-induced RNAi-mediated *prp8* knockdown is larval lethal.

**Fig. 5.**
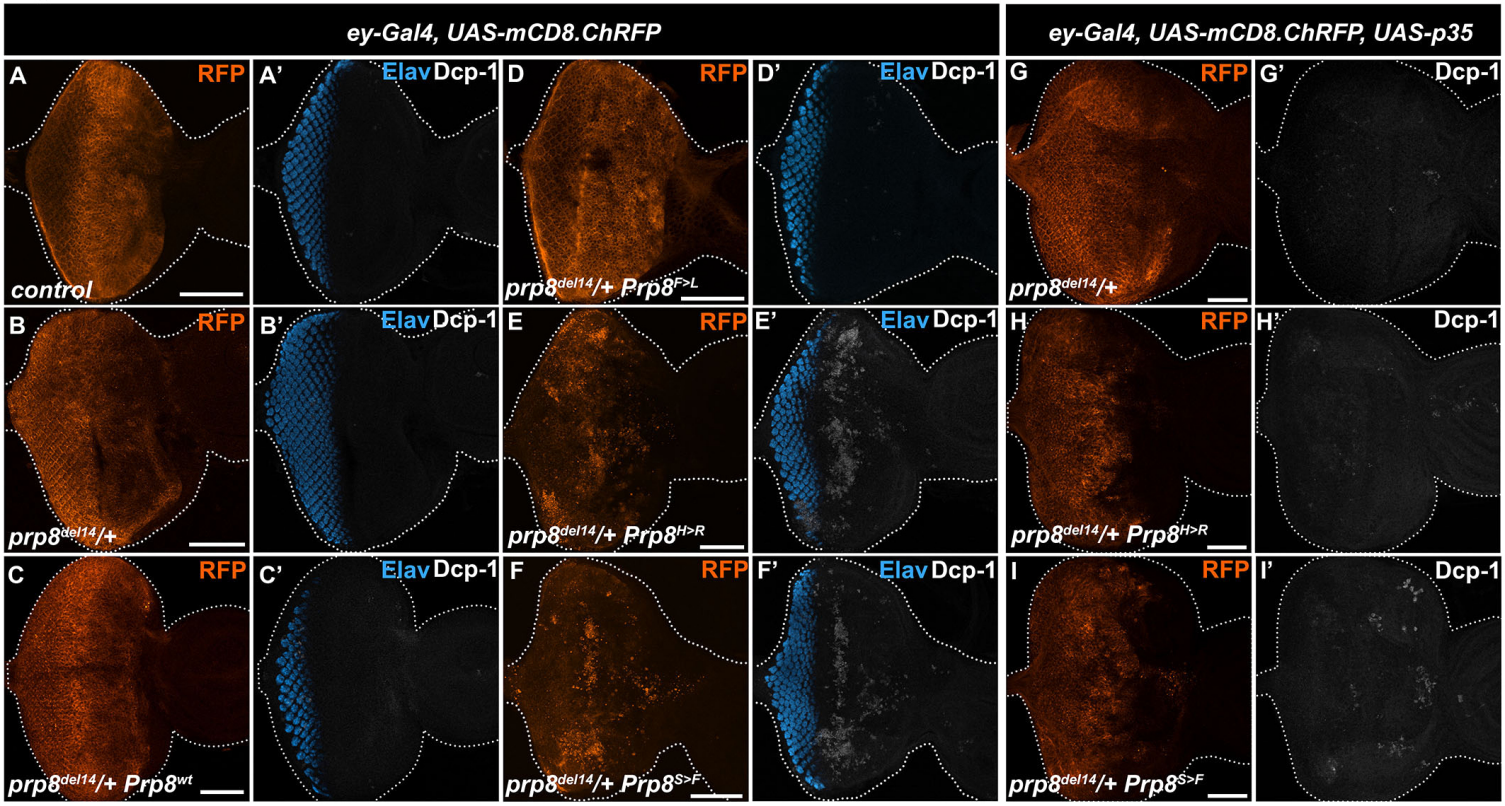
**Overexpression of Prp8^S>F^ and Prp8^H>R^ induces apoptosis in developing eye primordium.** (A–F) Compared with control (A) and *prp8^del14^*/+ heterozygous EADs (B), *ey*-specific overexpression of Prp8^H>R^ (E) and Prp8^S>F^ (F) but not Prp8^wt^ (C) and Prp8^F>L^ (D) induces apoptosis as shown by immunostaining against the *Drosophila* Death caspase 1 (Dcp-1). The majority of apoptotic cells localize anterior to the morphogenetic furrow outside of the differentiating ELAV-positive photoreceptor clusters. (G–I) Co-expression of p35 reduces the amount of Dcp-1 positive cells in Prp8^H>R^- and Prp8^S>F^-expressing *prp8^del14^*/+ heterozygous EADs (H,I) to levels observed in control (G). Representative micrographs are projections of multiple confocal sections showing EADs 7 days AEL. EAD outlines were generated based on DAPI signal. Scale bars: 50 µm.

To better reflect the heterozygous conditions of human RP patients, we overexpressed the Prp8^wt^, the asymptomatic Prp8^F>L^ and the two most pathogenic Prp8^H>R^ and Prp8^S>F^ variants in the heterozygous *prp8* mutant animals. To this end, we used the genome editing CRISPR/Cas9 technique to generate a *prp8^del14^* mutant allele that lacks the entire exon 14 encoding most of the C-terminal part of the protein (see Materials and Methods). Consistent with a vital role of Prp8 in pre-mRNA splicing, *prp8^del14^* homozygosity resulted in early embryonic and cell lethality (Fig. S5A,B,D) while heterozygous flies (*prp8^del14^/+*) were viable without any apparent developmental defects. Importantly, similar phenotypes have been reported for the *prp8^KG03188^* mutant allele harboring a P-element insertion within the 5′ UTR of the *prp8* gene (Fernandez-Espartero et al., 2018 and Fig. S5C,D). While *ey-Gal4*-driven expression of Prp8^wt^ and Prp8^F>L^ in *prp8^del14^* or *prp8^KG03188^* heterozygotes had no consequences (Fig. 4G–I,L, Fig. S5E–H,K), the expressivity of the phenotypic defects caused by Prp8^H>R^ and Prp8^S>F^ was higher (Fig. 4J–L, Fig. S5I–K) compared with the wild-type background (Fig. 4D–F). In addition to the aberrant shape and disarray of ommatidia (Fig. 4J,K, Fig. S5I,J), the adult eyes of *ey>Prp8^H>R^* and *ey>Prp8^S>F^* animals having only one wild-type *prp8* allele were also significantly smaller (Fig. 4Y, Fig. S5L). The reduced eye size indicated that the overexpression of the two pathogenic variants might induce cell death. Indeed, immunostaining with an antibody against the activated *Drosophila* Death caspase 1 (Dcp-1) revealed a marked enrichment of Dcp-1-positive cells within EADs overexpressing Prp8^H>R^ and Prp8^S>F^ (Fig. 5E,F) compared with the levels observed in Prp8^wt^, Prp8^F>L^ and control EADs (Fig. 5A–D). Importantly, co-expression of the baculovirus-derived pan-caspase inhibitor p35 but not the mock Flag tripeptide (Flag) was sufficient to alleviate the extent of cell death within *ey>Prp8^H>R^* and *ey>Prp8^S>F^ prp8^del14^/+* EADs (Fig. 5G–I, Fig. S6A-D) but also rescued the morphological abnormalities and size of the adult eyes (Fig. 4M–X,Z).

These results strongly argue for the dominant pathogenic effect of the two RP-Prp8 mutations, Prp8^H>R^ and Prp8^S>F^, on the normal development of the adult *Drosophila* eye. Expression of both mutant proteins induced cell death. Importantly, the majority of apoptotic cells localized anterior to the morphogenetic furrow of the developing eye primordium, whereas differentiated photoreceptors appeared more resistant to the toxicity of RP-Prp8 mutations or *prp8* downregulation.

### Cytoprotective and stress responses are hallmarks of the gene expression signature induced by RP-Prp8 mutations

**Fig. 6.**
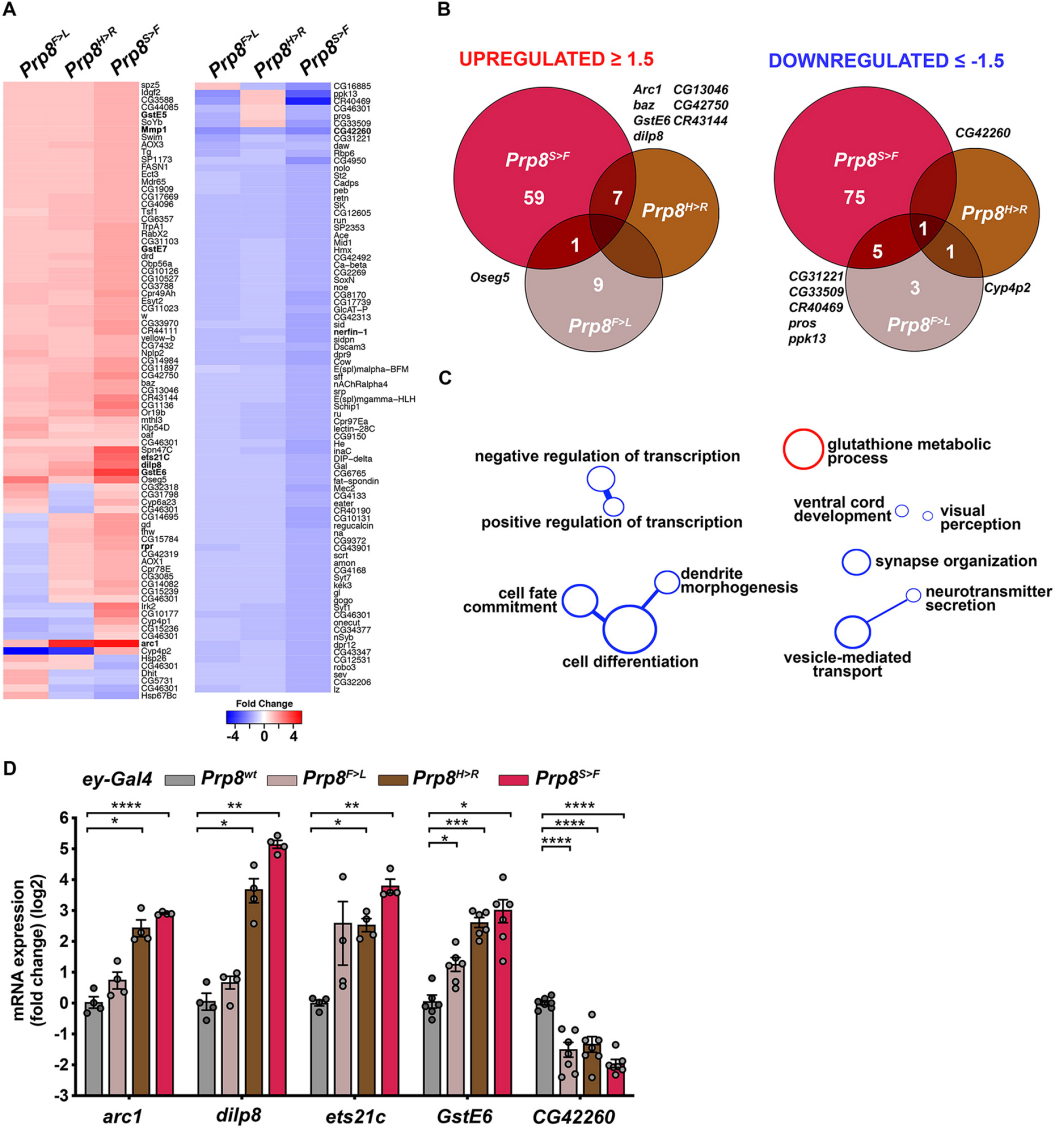
**RP-Prp8 mutant variants induce stress and cytoprotective gene expression program.** (A) The heatmap depicts genes significantly up- or downregulated following *ey*-specific overexpression of at least one of the RP-Prp8 mutant variants (|fold change|≥1.5, *P*<0.05) relative to those expressing Prp8^wt^. The expression changes of these transcripts in other experimental groups are also shown, but may not need to satisfy the criteria of *P*<0.05 significance. See Supplementary Dataset 1 for genes significantly changed. (B) Venn diagrams show overlap of genes significantly regulated (|fold change|≥1.5, *P*<0.05) in EADs expressing asymptomatic Prp8^F>L^ or the two toxic RP-Prp8 variants, Prp8^H>R^ or Prp8^S>F^. (C) Functional GO terms and clusters enriched among up- (red) and downregulated (blue) genes in *ey>Prp8^S>F^*-overexpressing EADs. (D) mRNA levels of stress-related and cytoprotective genes (*arc1*, *dilp8*, *ets21c*, *GstE6*) were significantly induced in response to *ey-*specific expression of Prp8^H>R^ and Prp8^S>F^ while *CG42260* related to neuronal function was downregulated relative to *ey>Prp8^wt^* samples. RT-qPCR data are means±s.d., *n*≥4. Statistical significance was determine using unpaired *t*-tests with Welch's correction assuming unequal variance; *P<0.05, **P<0.01, ***P<0.001, ****P<0.0001. The exact number and biological replicates per sample (*n*) and *P*-values are specified in Supplementary Dataset 2.

Given the differential phenotypic consequences of the RP-Prp8 mutations in the eye, we aimed to survey their impact on gene expression. To this end, we performed unbiased genome-wide transcriptome profiling of third instar larval EADs overexpressing Prp8^wt^, Prp8^F>L^, Prp8^H>R^ and Prp8^S>F^ proteins under the control of *ey-Gal4* in the wild-type background. The comparative RNA-seq analysis identified only a handful of genes that were differentially expressed (|fold change|≥1.5, *P*<0.05) (up/down) in response to Prp8^S>F^ (67/81 genes), Prp8^H>R^ (7/2 genes) and Prp8^F>L^ (10/10 genes) relative to EADs overexpressing Prp8^wt^ (Fig. 6A,B, Supplementary Dataset 1). While several upregulated genes were shared between the transcriptional profiles of the two pathogenic variants Prp8^H>R^ and Prp8^S>F^, the changes inflicted by Prp8^F>L^ were more distant (Fig. 6B). A gene ontology (GO) clustering analysis of transcripts upregulated in the Prp8^S>F^ dataset highlighted over-representation of genes linked to ‘Glutathione metabolism’ including three members of the glutathione S-transferase (GST) family *GstE6*, *GstE7*, *GstE5* (Fig. 6A,C, Supplementary Dataset 1). The induction of redox and detoxification genes positively correlated with an increased expression of several stress and damage response genes including the transcription factor *ets21c* (Külshammer et al., 2015; Mundorf et al., 2019), *matrix metalloprotease 1* (*Mmp1*) (Uhlirova and Bohmann, 2006) and a secreted *Drosophila insulin-like peptide 8* (*dilp8*) (Colombani et al., 2012; Garelli et al., 2012). In accordance with elevated apoptosis observed in Prp8^S>F^-overexpressing EADs (Fig. 5F, Fig. S6A), the pro-apoptotic gene *reaper* (*rpr*) was also induced (Fig. 6A, Supplementary Dataset 1). In contrast, downregulated genes were enriched for functions linked to ‘Regulation of transcription’, ‘Cell differentiation’ and GO terms describing neuronal morphogenesis and function including: ‘Dendrite morphogenesis’, ‘Synapse organization’, ‘Vesicle-mediated transport’, ‘Neurotransmitter secretion’ and ‘Visual perception’ (Fig. 6C, Supplementary Dataset 1). Importantly, the differential expression of several candidates was validated by RT-qPCR on independent samples (Fig. 6D). Compared with EADs overexpressing Prp8^wt^, *arc1*, *dilp8*, *ets21c* and *GstE6* transcripts were all increased in *ey>Prp8^H>R^* and *ey>Prp8^S>F^* samples, whereas the levels of *CG42260* were also significantly downregulated in *ey>Prp8^F>L^* EADs. CG42260 is the closest fly ortholog of human *CNGA3*, which encodes an α-subunit of the cone photoreceptor cGMP-gated cation channel. Interestingly, mutations in *CNGA3* have been linked to total colour blindness, also referred to as rod monochromacy (RM) or complete achromatopsia, a rare, autosomal recessive inherited and congenital disorder (Kohl et al., 1998; Wissinger et al., 1998).

Taken together, our genome-wide transcriptome profiling revealed that the developing eye primordium induces expression of damage, stress and detoxification genes in response to the toxic RP-Prp8 protein variants, likely in an attempt to alleviate the damage and regain homeostasis at the expense of normal neurogenic development and differentiation. Despite a marked difference in the number of dysregulated genes, the genetic programme triggered by Prp8^F>R^ and Prp8^S>F^ shares common features ultimately resulting in similar phenotypic outcomes.

### Pathogenic RP-Prp8 mutations cause redox imbalance upstream of cell death

**Fig. 7.**
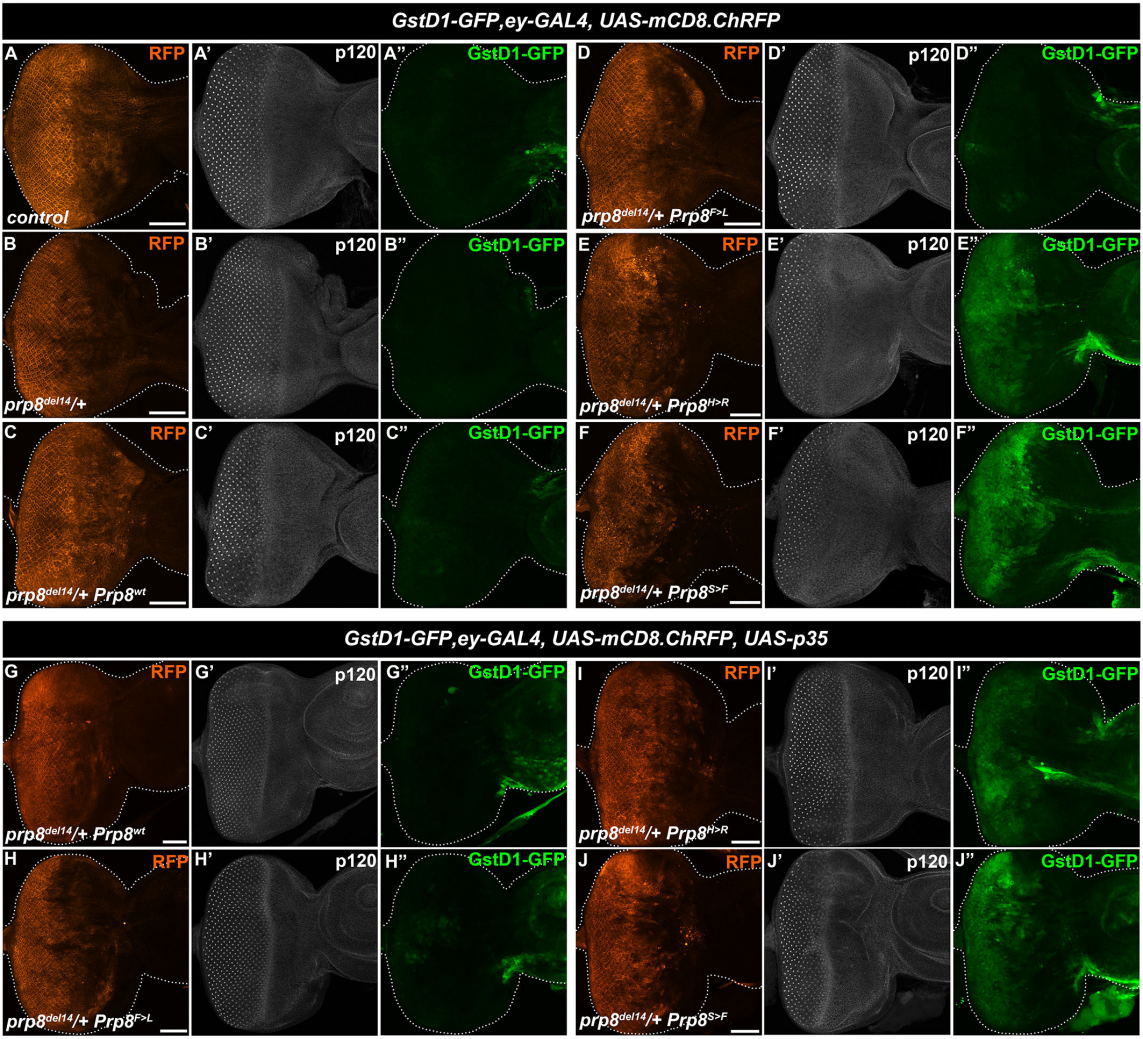
**Differentiated as well as uncommitted cells induce cytoprotective response to the Prp8^S>F^ and Prp8^H>R^ mutant variants.** (A–F″) *Ey*-specific overexpression of Prp8^H>R^ (E) and Prp8^S>F^ (F) markedly induced the *GstD1-GFP* reporter activity in EADs of *prp8^del14^*/+ heterozygous larvae compared to the background levels in controls (A,B) and EADs expressing Prp8^wt^ (C) and Prp8^F>L^ (D). Note the enhancement of the *GstD1-GFP* signal in both differentiating cells posterior as well as uncommitted epithelial cells anterior to the morphogenetic furrow expressing Prp8^H>R^ (E″) and Prp8^S>F^ (F″). Morphogenetic furrow and differentiated ommatidia clusters are visualized by immunostaining against p120-catenin. (G–J″) The *GstD1-GFP* induction in Prp8^H>R^- and Prp8^S>F^-expressing *prp8^del14^*/+ heterozygous EADs (I″,J″) was not inhibited by co-expression of p35. Note that *ey-*driven p35 expression did not interfere with the endogenous *GstD1-GFP* reporter activity in the antenna (G″–J″). Representative micrographs are projections of multiple confocal sections showing EADs 7 days AEL. Images were acquired with the same intensity settings. Disc outlines were generated based on DAPI signal. Scale bars: 50 µm.

GSTs are among the best-known phase II detoxifying enzymes that conjugate glutathione to harmful hydrophobic electrophiles including xenobiotics and activated metabolites. The upregulation of several of the GST genes in *ey>Prp8^H>R^* and *ey>Prp8^S>F^* samples indicated that EAD cells suffer from the breakdown of the intracellular redox homeostasis. To support the notion, we took advantage of the transgenic *GstD1-GFP* reporter (Sykiotis and Bohmann, 2008), which has been used to detect changes in the cellular redox state. The *GstD1-GFP* reporter contains consensus binding motifs for the transcription factors such as Nrf2 and Foxo which act downstream of signalling pathways activated by reactive oxygen species (ROS). Indeed, we found a marked upregulation of the *GstD1-GFP* reporter (Sykiotis and Bohmann, 2008) in Prp8^H>R^- and Prp8^S>F^-expressing EADs (Fig. 7E,F, Fig. S6A) compared to controls (Fig. 7A). Interestingly, both differentiated and uncommitted, epithelial cells within the *ey* domain induced the detoxification response. In contrast, neither *prp8* heterozygosity nor Prp8^wt^ or Prp8^F>L^ *ey*-specific overexpression activated the *GstD1-GFP* reporter (Fig. 7B–D). Intriguingly, inhibiting apoptosis by co-expression of p35 did not reduce the upregulation of *GstD1-GFP* in *ey>Prp8^H>R^* and *ey>Prp8^S>F^ prp8^del14^* heterozygous EADs (Fig. 7G–J, Fig. S6B). Of note, co-expression of a mock Flag tripeptide had no impact on *GstD1-GFP* reporter activity (Fig. S6C,D). These results demonstrate that although blocking apoptosis prevented alterations to the overall size and morphology of the adult eyes, it did not alleviate the redox imbalance caused by the presence of the toxic RP-Prp8 mutant proteins.

## DISCUSSION

Recent progress in the identification of genetic causes of RP highlights malfunction of the U4/U6.U5 tri-snRNP components of the spliceosome in the etiology of this blinding disorder, and sparked significant interest in generation of animal models to understand how aberrant splicing contributes to the tissue-specific pathophysiology. While vertebrate models, including mice and zebrafish, are well suited for investigation of splicing factor RP pathogenesis (Graziotto et al., 2011; Keightley et al., 2013), the large number of mutations in different U4/U6.U5 tri-snRNP components makes the generation of mutant, knock-in or transgenic lines time-consuming and cost-ineffective. Over time, the *Drosophila melanogaster* model has proven successful in unravelling the disease biology of six different RP classes, including RP4, RP11, RP12, RP14, RP25 and RP26 (Lehmann et al., 2019). Moreover, it has been instrumental in deciphering how genetic variation influences the phenotypic variability frequently observed in RP individuals carrying the same pathogenic mutation (Chow et al., 2016).

Here, we establish the *Drosophila* model of human RP13 caused by mutations in Prp8, the key component of the U4/U6.U5 tri-snRNP and the catalytic core of the spliceosome. We demonstrate the versatility of the fly model to screen and compare the tissue-specific impact of different RP-Prp8 mutations and its suitability to untangle the genetic and molecular mechanisms underlying their pathogenesis. We show that targeted expression of nine different RP-Prp8 mutant proteins in postmitotic, endoreplicating cells of a larval prothoracic gland delayed the timing of the larval to pupal transition. The expressivity of the phenotype varied among the different mutations, with Prp8^H>R^ and Prp8^S>F^ being the most detrimental, the latter causing a complete developmental arrest. The severity of the developmental phenotype induced by Prp8^H>R^ and Prp8^S>F^ correlated with deterioration of the PG morphology and aberrant splicing of the *spok* pre-mRNA. Interestingly, neither of the tested RP-Prp8 mutant proteins produced a visible phenotype when overexpressed in differentiated photoreceptors under the control of the late-acting *GMR-Gal4* driver. In contrast, the two mutations with the most severe effect in the PG, Prp8^H>R^ and Prp8^S>F^, interfered with the normal development of the adult eye when their expression was triggered early in the eye/antenna primordium compared with asymptomatic expression of wild-type Prp8 and the seven other RP-Prp8 mutations. The Prp8^H>R^- and Prp8^S>F^-induced phenotypes, including reduced size and disturbed adult eye morphology, were further enhanced by *prp8* heterozygosity. As we did not observe differences in the stability of the individual transgenes, these findings support the notion that the phenotypic consequences of RP-Prp8 mutations result from dominant-negative or gain-of-function mechanisms, rather than haploinsufficiency (Graziotto et al., 2011). Remarkably, the pathogenicity of the RP-Prp8 mutations in the *Drosophila* RP13 model correlates with severity of growth defects observed in yeast, as well as strength of disease phenotypes in patients carrying the equivalent mutations. In yeast, Prp8^H>R^, Prp8^S>F^ and Prp8^H>P^ mutations exhibited the most profound growth defects (Boon et al., 2007; Maeder et al., 2009; Mozaffari-Jovin et al., 2013). Human carriers of Prp8^H>R^ and Prp8^H>P^ mutations suffer from earlier onset of night blindness, more severe prognosis for visual acuity and earlier loss of central vision when compared with Prp8^R>K^ patients (Escher et al., 2018; Towns et al., 2010). It remains to be determined why Prp8^H>P^ mutation, affecting the same amino acid residue as Prp8^H>R^, appears asymptomatic in the *Drosophila* RP13 model. The molecular mechanisms underlying how individual RP-Prp8 mutations cause adRP remain an intriguing question. All nine tested mutations cluster in the C-terminal Jab1/MPN domain, which is essential for interaction with Brr2 and timely regulation of its ATP-dependent helicase activity (Boon et al., 2006; Grainger and Beggs, 2005). Studies in the budding yeast and HeLa cells revealed that some of the RP-Prp8 mutants (e.g. Prp8^S>F^ and Prp8^H>R^) cannot efficiently incorporate into the maturing U5 snRNP, while others (e.g. Prp8^F>L^ Prp8^Y>N^) permit U5 and tri-snRNP assembly but compromise U4/U6 unwinding (Boon et al., 2007; Malinová et al., 2017). Although difficult to uncouple, both defects ultimately lead to the scarcity of mature particles, which might compromise the splicing efficiency, specificity and/or fidelity in tissues with a high demand for general pre-mRNA splicing or processing of specific transcripts. However, it is also plausible that other mechanisms including splicing-independent roles of Prp8 could contribute to RP-Prp8 pathogenesis. One such mechanism could be proteotoxic stress caused by overwhelmed chaperone and/or proteasome machineries with folding-defective RP-Prp8 mutant proteins or immature snRNPs (Růžič ková and Staněk, 2017). In support of this notion, experiments in HeLa cells revealed enhanced binding of Prp8^H>R^, Prp8^S>F^ to AAR2, a crucial chaperone of Prp8, compared with the wild-type protein and other assessed RP-Prp8 mutant variants. Intriguingly, the same mutants also exhibited a stronger association with components of the RT2P complex, which acts as a co-chaperone of Heat shock protein 90 (HSP90) and controls biogenesis of multi-subunit machines including the small nucleolar ribonucleoproteins (snoRNPs), nutrient sensing mTORC1 and RNA polymerase II (Pol II) (Malinová et al., 2017; von Morgen et al., 2015). In this regard, it is also interesting to note that Prp8 is an inactive deubiquitinating enzyme with ubiquitin binding activity within the MPN/JAB domain (Bellare et al., 2008; Grainger and Beggs, 2005; Komander et al., 2009; Pena et al., 2007). Studies in yeast using ubiquitin mutants or ubistatins revealed a direct role of ubiquitin in U4/U6 unwinding (Bellare et al., 2008). Whether RP-Prp8 mutations alter ubiquitin binding remains to be determined. Finally, a growing body of evidence suggests reciprocal coupling of RNA processing and transcription, as splicing factors have been implicated in the regulation of transcription initiation, elongation rate of Pol II and the choice of transcriptional start sites (Fiszbein et al., 2019; Lin et al., 2008; Maslon et al., 2019). Splicing factor malfunction can thus manifest in a noticeable shift in gene expression profile rather than obvious splicing defects.

Despite the genetic heterogeneity of RP, there are extensive data from animal models and RP patients implicating oxidative damage among the common drivers of cone photoreceptor cell death. Sampling of aqueous humour invariably detected excessive protein carbonylation and a lower ratio of reduced to oxidized glutathione, which are considered among the major signs of oxidative damage and indicators of antioxidant defence system failure. Administration of drugs or gene transfer that reduce oxidative stress have been shown to promote cone survival and maintenance of cell function (Campochiaro and Mir, 2018). Consistently, our transcriptome profiling revealed an upregulation of cytoprotective, stress, damage response and apoptotic genes in EADs expressing the pathogenic Prp8^S>F^ and Prp8^H>R^ mutants at the expense of genes required for neurogenesis and neuron function. With the help of the transgenic *GstD1-GFP* reporter, we further determined that both differentiated photoreceptors and actively cycling epithelial cells upregulated the phase II detoxifying programme. While differentiated cells survived, uncommitted eye progenitors underwent apoptosis. Interestingly, the phenomenon of acquired apoptosis resistance of terminally differentiated cells without regenerative potential in *Drosophila* has been attributed to the epigenetic silencing of major pro-apoptotic gene loci during development (Zhang et al., 2008). Blocking effector caspases rescued the cell loss, allowing flies to eclose with normally sized eyes, yet the cells still suffered from redox imbalance. What triggers the stress and antioxidant defence programmes and whether the scavenging of reactive oxygen species might be effective in alleviating the RP-Prp8 toxicity remain interesting avenues of research for future investigations.

## MATERIALS AND METHODS

### Fly stocks

The following *Drosophila* strains were used: (a) *w^1118^*, (b) *phm-Gal4* (RRID:BDSC_80577) (c) *ey-Gal4* (RRID:BDSC_5534), (d) *GMR-Gal4* (RRID:BDSC_1104), (e) *UAS-prp8^RNAi^* (VDRC, 18565), (f) *dpp-Gal4* (RRID: BDSC_7007), (g) *UAS-Flag::Prp8^wt^* (Claudius et al., 2014), (h) *FRT82B* (RRID: BDSC_2035), (i) *prp8^del14^/CyO, act-GFP JMR1* (this study), (j) *UAS-p35* (RRID: BDSC_5072), (k) *y,v, nos-phiC31\int.NLS; attP2* (RRID:BDSC_25710), (l) *UAS-myr-mRFP* (RRID:BDSC_7118), (m) *UAS-mCD8.ChRFP* (RRID:BDSC_27391), (n) *UAS-mCD8::GFP.L* (RRID:BDSC_5130) (o) *eyFLP, act>y+>Gal4, UAS-GFP; FRT82B tubGal80* (Pagliarini and Xu, 2003), (p) *FRT42D* (RRID:BDSC_1802), (q) *eyFLP; FRT42D tub-Gal80/CyO; act>y+>Gal4, UAS-GFP/TM6B att.* (this study), (r) *Act5C-cas9, Lig4[169]* (RRID:BDSC_54590) (Zhang et al., 2014), (s) *GstD1-GFP* (Sykiotis and Bohmann, 2008) (t) *UAS-Flag* (this study), (u) *prp8^KG03188^* (RRID:BDSC_13006).

All crosses were set up and maintained at 25°C, unless specified otherwise, on a diet consisting of 0.8% agar, 8% cornmeal, 1% soymeal, 1.8% dry yeast, 8% malt extract and 2.2% sugar-beet syrup, which was supplemented with 0.625% propionic acid and 0.15% Nipagin. *Gal4* driver lines crossed to *w^1118^* and *FRT42D* served as controls for experiments performed in the wild-type and *prp8* (*prp8^del14^* or *prp8^KG03188^*) heterozygous background, respectively. Overexpression of the Flag tripeptide was used as a mock control to exclude saturation of the *Gal4* driver. All stocks are listed in Table S1.

### Generation of plasmids and transgenic lines expressing RP-Prp8 variants

The alignment of human (Q6P2Q9), *Drosophila* (A1Z8U0) and *S. cerevisiae* (P33334) Prp8 proteins was created using MUSCLE multiple sequence alignment software (Edgar, 2004). The amino acid identity was determined using Clustal Omega (Sievers et al., 2011). Coding sequence of *Drosophila melanogaster prp8* (*CG8877*) was amplified from cDNA using the Phusion polymerase (New England Biolabs) and cloned into the *pENTR4* vector (Invitrogen). Nine different RP mutations were introduced using the Quick Change Lightning site directed mutagenesis kit (Agilent). All primers for mutagenesis are listed in Table S3. The N-terminal Flag-tag was added by LR Clonase II-mediated recombination (Invitrogen) into the pTFW vector (DGRC). Following restriction with SphI and NotI, the UAS-Flag::Prp8 cassettes were inserted into the pattB vector backbone (DGRC, Bischof et al., 2007). The non-tagged variants were generated by cutting out the Flag-tag coding sequence from the *pUAST-attB-Flag::Prp8* vectors with AgeI and AvrII and re-ligating the Klenow-filled blunted ends. All vectors and plasmids are listed in Table S2.

Transgenic fly lines allowing overexpression of untagged wild-type and RP-Prp8 mutant variants (S2178F/Prp8^S>F^, P2361T/Prp8^P>T^, F2374L/Prp8^F>L^, H2369P/Prp8^H>P^, H2369R/Prp8^H>R^, H2370G/Prp8^H>G^, H2370K/Prp8^H>K^, R2370S/Prp8^R>S^, Y2395N/Prp8^Y>N^) were established by PhiC31 integrase-mediated transgenesis of the respective *pUAST-attB* vectors into *attP-9A* site (99F8) (BestGene Inc.). The N-terminally-tagged RP-Prp8 constructs (Flag::Prp8^S>F^, Flag::Prp8^P>T^, Flag::Prp8^F>L^, Flag::Prp8^H>P^, Flag::Prp8^H>R^, Flag::Prp8^H>K^, Flag::Prp8^R>S^) were integrated into the *attP2* site (68A4) (BestGene Inc.). The UAS-Flag transgenic flies expressing the Flag tripeptide were obtained by standard P-element-mediated germline transformation of pTFW plasmid into *w^1118^ Drosophila* embryos (BestGene Inc). All stocks are listed in Table S1.

### Generation of EAD genetic mosaics

The mosaic analysis with a repressible cell marker (MARCM) technique (Lee and Luo, 2001) with *eyFLP; act>y+>Gal4, UAS-GFP; FRT82B tub-Gal80* or *eyFLP; FRT42D tub-Gal80/CyO; act>y+>Gal4, UAS-GFP/TM6B* flies was used to generate genetically defined clones within the EADs as described in Mundorf and Uhlirova (2016).

### Generation of *prp8* mutant fly line

The *prp8* mutant line was obtained by CRISPR-Cas9 genome editing. Two guide RNAs (sgRNA), targeting intron 12 and 3′-UTR of *Drosophila prp8* gene (Table S3) were cloned into the pCFD4-U6:1_U6:3 tandem gRNAs vector (Port et al., 2014). Fragments for cloning were amplified using Phusion HS II polymerase (Thermo Scientific), with the vector itself serving as the PCR template. The CFD4-U6:1_U6:3 tandem gRNAs-Prp8 construct was subsequently integrated into the *attP2* site on the third chromosome (68A4) using PhiC31 integrase-mediated transgenesis. Upon crossing to *Act5C-cas9, Lig4[169]* (Zhang et al., 2014), progeny was collected, balanced and screened by PCR to detect the deletion. The recovered *prp8^del14^* allele contains a deletion of 2998 bp encoding a truncated Prp8 protein which lacks 981 amino acids at the C-terminus.

### Generation of *Drosophila* Prp8 antibody

Polyclonal antibodies specific to the C-terminal part of *Drosophila* Prp8 protein (CTD) including the MPN/Jab1 and Tail domain were raised by immunizing two rabbits (Eurogentec) with a 6xHis-tagged Prp8 polypeptide (amino acids 2092–2341). The antigen was expressed from the Gateway pDEST17 vector (Thermo Scientific) in *E. coli* BL21(DE3)pLysS bacterial strain and purified under denaturing conditions using immobilized metal affinity chromatography (IMAC).

### Cell culture

*Drosophila* S2 cells were cultured at 25°C in Shield and Sang M3 insect medium (Sigma-Aldrich) supplemented with 8% FBS (Merck-Biochrom). Cells were transfected in 6-well plates on glass coverslips, in serum-free medium with the desired plasmids using TransIT-Insect (Mirus Bio) according to manufacturer's instructions. Expression of UAS-driven constructs was induced by co-transfection with a pAW-GAL4 plasmid expressing Gal4 under an *Actin5C* promoter. Transfection efficiency was monitored by co-transfection of pIE-EGFP plasmid. Cells were processed 30 h post-transfection.

### SDS-PAGE and western blot

Eye/antennal discs (>40 EADs/replicate/genotype) were dissected 7 days after egg-laying (AEL) in 1× PBS, and immediately lysed in 50 mM Tris-HCl (pH 7.8), 150 mM NaCl, 1 mM EDTA (pH 8.0), 1% Triton X-100, 0.01% Igepal and protease inhibitors (Roche Applied Science). After centrifugation, protein concentration in the supernatant was determined using Bradford assay reagent (Bio-Rad) according to the manufacturer's instructions. Samples were denatured by boiling in Laemmli buffer containing 2.5% β-mercaptoethanol for 5 min at 95°C. Equal amounts of total protein were loaded on a 10% polyacrylamide gel. After SDS-PAGE, proteins were detected by immunoblotting with mouse anti-Flag M2 (1:1000, Sigma-Aldrich) (RRID: AB_262044), rabbit anti-dPrp8-CTD (1:1000, this study, Eurogentec), and mouse anti-ATP5α (1:2000, Abcam) (RRID:AB_301447) followed by incubation with the corresponding HRP-conjugated secondary antibodies. Chemiluminescence was detected with ImageQuant LAS4000 reader (GE Healthcare). ImageJ (https://fiji.sc/) (RRID: SCR_003070) was used to determine the intensities of Prp8, Flag and ATP5α signals using the built-in Gel analysis tools. The intensities of a loading control (ATP5α) in individual western blot replicates were compared to establish a normalization factor which was used to correct the intensities of Prp8 or Flag signals. For each biological replicate, the relative expression of Prp8 or Flag was normalized to the average expression across all samples within a replicate.

### Cell and tissue staining

EADs, wing discs and PGs dissected from third instar *Drosophila* larvae (7 days AEL) and *Drosophila* S2 cells were fixed for 25 min with 4% paraformaldehyde in PBS containing 0.1% Triton X-100 (PBS-T) and washed 3 times with PBS-T. After blocking in 0.3% BSA in PBS-T samples were incubated overnight at 4°C with the following primary antibodies at the indicated dilutions: guinea pig anti-Spok (1:1000, Ono et al., 2006), rabbit anti-Dcp-1 (1:500, Cell Signaling Technology) (RRID:AB_2721060), rat anti-Elav (1:500, DSHB) (RRID: AB_528217), mouse anti-p120 (1:300, DSHB) (RRID: AB_2088073), rabbit anti-GFP (1:300, Thermo Scientific) (RRID:AB_2536526), rabbit-anti-dPrp8-CTD (1:500, this study, Eurogentec), mouse anti-Flag M2 (1:500, Sigma-Aldrich) (RRID: AB_262044). After washing, the samples were incubated with the corresponding Alexa Fluor 488- or CY5-conjugated secondary antibodies (Thermo Scientific or Jackson ImmunoResearch) for 2 h at room temperature and counterstained with DAPI (1 µg/ml, Carl Roth GmbH) to visualize nuclei. Tissues were mounted on glass slides in Dabco-Mowiol (Sigma-Aldrich).

### Image acquisition and processing

Confocal images and stacks were acquired with Olympus FV1000 confocal microscope equipped with 20× UPlan S-Apo (NA 0.85), 40× UPlan FL (NA 1.30) and 60× UPlanApo (NA1.35) objectives. Maximum Z-projections were generated from a maximum of seven consecutive sections taken at 0.4 µm steps using Fluoview 1000 Software (Olympus) (RRID: SCR_014215) and ImageJ (https://fiji.sc/) (RRID: SCR_003070). Final image processing including panel assembly, brightness and contrast adjustments were performed in Adobe Photoshop CC (Adobe Systems, Inc.) (RRID: SCR_014199). White outlines of the EADs and ring glands shown in figures were drawn based on DAPI staining and GFP signal, respectively. For measurements of the adult eyes, 3- to 8-day-old male flies were collected from multiple vials of the same cross and the genotypes were concealed for sample preparation and data evaluation. Z-stacks of left eyes were taken by a single person using an Olympus SZX16 fluorescent stereomicroscopes equipped with a DP72 CCD camera under the same magnification. Images were processed with cellSens 1.1 Software (Olympus) (RRID: SCR_014551). Outlines of the adult eyes (area containing ommatidia) were prepared in Adobe Photoshop CS5.1 (Adobe Systems, Inc) (RRID:SCR_014199) with the magnetic or polygonal lasso tool and superimposed to represent deviations in eye morphology within and among genotypes. Statistical significance was determined by ordinary one-way ANOVA with Tukey's multiple comparisons test in GraphPad Prism (RRID:SCR_002798). For quantification of the GFP-positive clonal volume, confocal Z-stacks spanning the columnar epithelia of mosaic EADs were imported into ImageJ (https://fiji.sc/) (RRID: SCR_003070). After thresholding, the individual slices were converted to binary images and the outlines selected. The ratio of GFP and DAPI was determined using the 3D manager plug-in. The same macros were applied to all samples; all macros used are available upon request.

### Pupation analysis

Female flies (*phm>mCD8::GFP*) crossed to the males carrying the different *pUAST-attB*-*Prp8* transgenes were allowed to lay eggs for 24 h. Early third instar larvae (≥25) were transferred to fresh vials. Two vials for each genotype were kept at 25°C and the pupae were counted twice a day. The entire experiment was repeated twice. Statistical significance among genotypes was calculated with a Log-rank (Mantel–Cox) test. The curves prepared with GraphPad Prism represent one of two independent experiments. The number of flies per experiment and genotype (*n*) and *P*-values are specified in Supplementary Dataset 2.

### RNA extraction, cDNA synthesis and qPCR

To assess the splicing of *spok* pre-mRNA in the PG, third instar larvae (7 days AEL) (8 larvae per replicate, *n*≥4) of the respective *phm-Gal4* genotype were collected. For RNA-seq and RT-qPCR, EADs were dissected from third instar larvae (7 days AEL) (∼100 discs per replicate, *n*≥3) overexpressing the RP-Prp8 variants under the *ey-Gal4* driver. RNA was extracted using the standard protocol with Tri-Reagent (Sigma-Aldrich) and DNase I treatment (Ambion) (Mundorf and Uhlirova, 2016). cDNA was synthesized with Superscript III reverse transcriptase (Thermo Scientific) and random hexamer primers from 2 µg (whole larvae) or 600 ng (dissected EADs) of total RNA. A 1:10 dilution of the cDNA was used as the template for qPCR, performed in triplicates with GoTaq qPCR Master Mix (Promega) on a CFX96 real-time PCR system (Bio-Rad). RT-qPCR primers (Table S3) were designed to anneal at 62°C. Data were normalized to the expression levels of *rp49* transcript, and fold-changes calculated using the ΔΔCt method (Livak and Schmittgen, 2001). Statistical analysis and graphical representation of the data was performed with GraphPad Prism. An unpaired *t*-test with Welch's correction was used to determine statistical significance for changes in gene expression. The sample sizes for RT-qPCR were determined as described previously (Mundorf et al., 2019).

### RNA-seq and data analysis

Total RNA extracted from dissected EADs (*n*=3/genotype) was used for library preparation using the Illumina TruSeq kit according to manufacturer's instructions. The libraries were paired end sequenced on the Illumina HiSeq 2000, at 75 bp read length with >45 million reads per library. The data were processed with an in-house RNA-Seq analysis pipeline (QuickNGS). In short, initial quality check was performed using FastQC (Supplementary Dataset 1), and the reads were aligned to the *Drosophila* reference genome BDGP Release 6 (dm6) using Tophat2 v.2.0.10 (Kim et al., 2013). The transcriptome was assembled with Cufflinks v.2 2.1.1 (Trapnell et al., 2010). Differential gene expression and exon usage was determined by DESeq2 (Anders and Huber, 2010) and DEXSeq (Anders et al., 2012), respectively. Genes with |fold change|≥1.5 and *P*<0.05 were considered as significantly up- or downregulated compared with the EADs expressing Prp8^wt^. The FlyBase Gene Ontology (GO) terms were used for functional annotation. The GO category enrichment analysis of differentially regulated genes was performed with DAVID considering the GO_BP_FAT ontology (http://david.abcc.ncifcrf.gov/) (Huang et al., 2009a,b). The GO term clustering and visualization was performed with the help of REViGO (http://revigo.irb.hr/) (RRID: SCR_005825) (Supek et al., 2011).

## Supplementary Material

Supplementary information
