# Supplementary material for: A Drosophila model to study retinitis pigmentosa pathology associated with mutations in the core splicing factor Prp8
Source: Dis Model Mech. 2020 Jun 26;13(6):dmm043174. doi: 10.1242/dmm.043174 (PMC7328144; doi:10.1242/dmm.043174)
Supplement: Supplementary information [file dmm-13-043174-s1.pdf]

A *Drosophila* model to study retinitis pigmentosa pathology associated with mutations in the core splicing factor Prp8

Dimitrije Stanković [1], Ann-Katrin Claudius[1], Thomas Schertel[1], Tina Bresser[1], Mirka Uhlirova[1,2*]

Figure S1

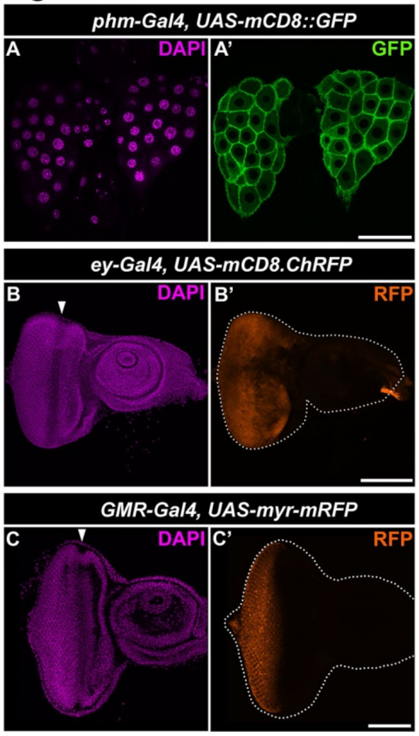

Fig. S1. Expression patterns of Gal4 drivers used in the study.

(**A**) *phm-Gal4* driver is active in the *Drosophila* prothoracic gland as depicted by GFP expressed from the UAS-based transgene. (**B**) *ey-Gal4* is active in the eye primordium of the larval EAD in cells both anterior and posterior to the morphogenetic furrow (arrowhead) as depicted by RFP expression. (**C**) The late acting *GMR-Gal4* driver expresses in cells posterior to the morphogenetic furrow (arrowhead) of larval EAD as indicated by the RFP signal. (A-C) Micrographs show projections of multiple confocal sections of respective tissues dissected from third instar larvae 7 days AEL. Scale bars: 50 μm (A) and 100 μm (B, C).

Disease Models & Mechanisms • Supplementary information

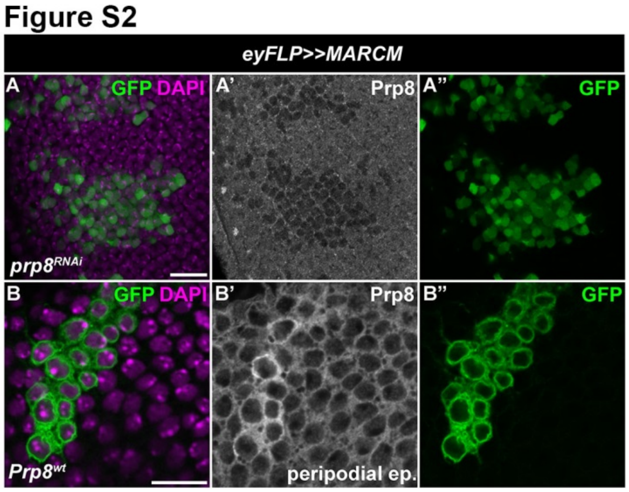

Fig. S2. Prp8 antibody detects endogenous as well as transgenic *Drosophila* Prp8 proteins. (**A-B**) *eyFLP*-mediated mitotic recombination was used to generate clones (GFP) in EADs of the indicated genotypes. Immunostaining of EADs with the self-made antibody directed against the CTD of the *Drosophila* Prp8 protein revealed reduction of Prp8 levels in clones where *prp8* transcript was knocked down by RNAi relative to non-clonal tissue (A'). In contrast, Prp8 levels were increased in cells overexpressing Prp8^{wt} (B'). Note the cytoplasmic localization of the endogenous and transgenic Prp8 proteins (B'). Discs were counterstained with DAPI. Micrographs are single confocal slices of EADs 7 days after egg laying. Scale bars: 10 μm.

Disease Models & Mechanisms • Supplementary information

Figure S3

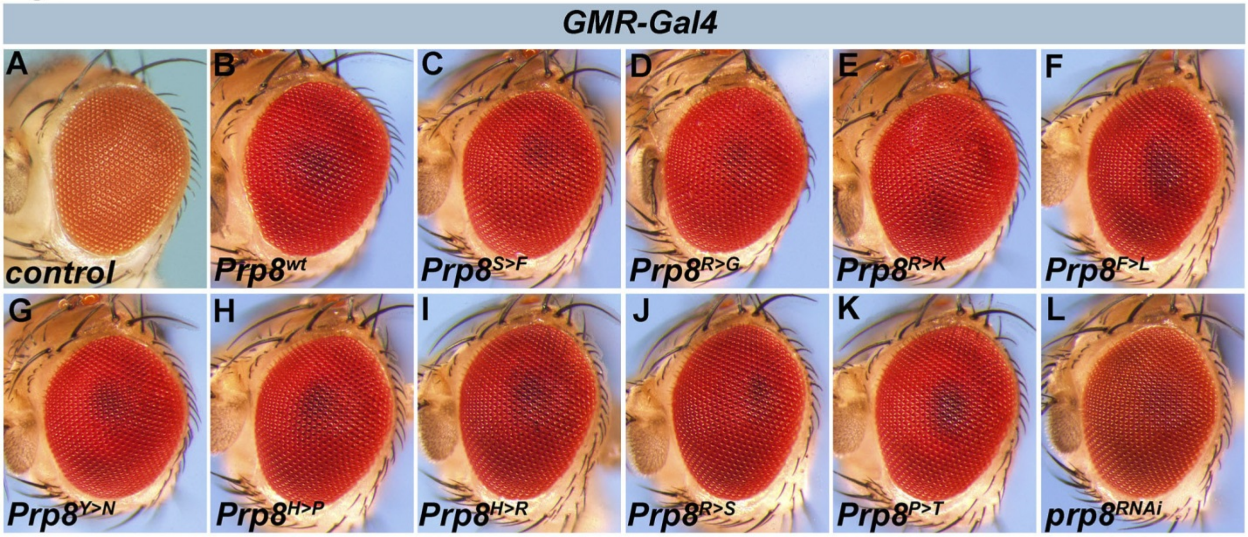

Fig. S3. Expression of RP-Prp8 variants under the *GMR-Gal4* driver is asymptomatic.

(**A-L**) Overexpression of wild-type Prp8 (B) or any of the nine different RP-Prp8 mutant variants (C-K) or RNAi-mediated knockdown ($prp8^{RNAi}$) (L) using the late-acting *GMR-Gal4* driver did not have any detrimental effect on the development of the adult eye and were comparable to control (A).

Disease Models & Mechanisms • Supplementary information

Figure S4

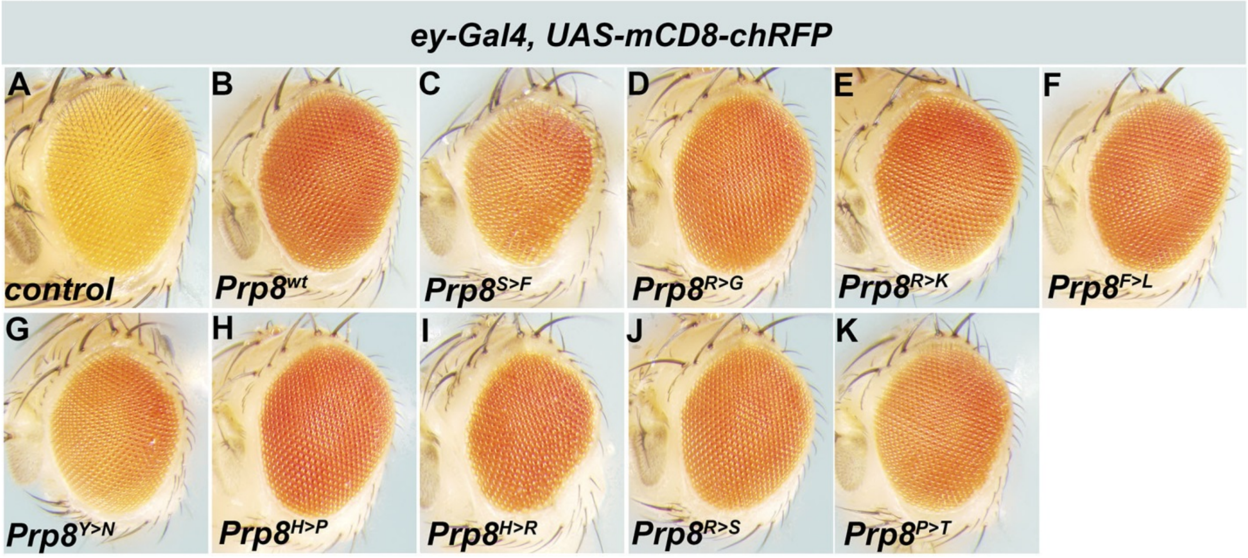

Fig. S4. Differential impact of early induction of RP-Prp8 mutations on the adult eye development.

(**A-K**) Expression of $Prp8^{S>F}$ (C) and $Prp8^{H>R}$ (I) in the EADs using the early acting *ey-Gal4* driver resulted in rough and irregularly shaped adult eyes compared to control (A) and those expressing $Prp8^{wt}$ (B) or other RP-Prp8 variants (D-H, J, K).

Disease Models & Mechanisms • Supplementary information

Figure S5

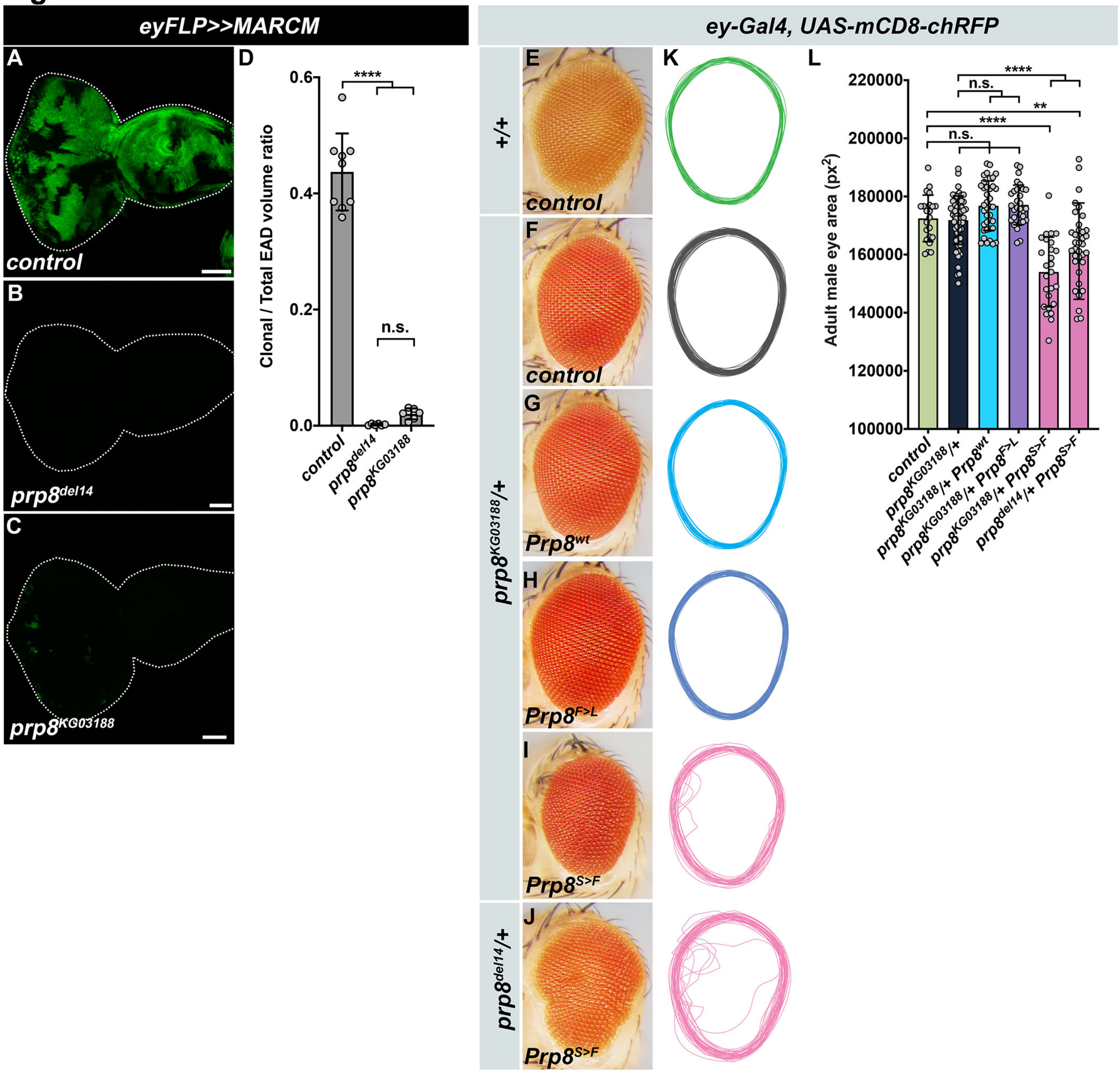

Fig. S5. Prp8 loss is incompatible with cell survival and halving the *prp8* gene dose enhances RP-Prp8-induced phenotypes.

(**A-D**) eyFLP-mediated mitotic recombination was used to generate homozygous clones (GFP) in EADs of the indicated genotypes. In contrast to control (A), cells homozygous for the $prp8^{del14}$ deletion allele (B) or the $prp8^{KG03188}$ insertion allele (C) do not survive and do not contribute to the total volume of the EAD tissue (D). (**E-L**) RP-Prp8 mutant variants were expressed in $prp8^{KG03188}$/+ heterozygous

Disease Models & Mechanisms • Supplementary information

background using the *ey-Gal4* driver. In contrast to controls (w^{1118} +/+, E; $prp8^{KG03188}$/+, F) and asymptomatic overexpression of $Prp8^{wt}$ (G) and $Prp8^{F>L}$ (H), $Prp8^{S>F}$ disrupts morphology and reduces size of the adult eyes (I, L) mimicking phenotypes observed when overexpressed in $prp8^{del14}$/+ heterozygotes (J, L). (D, L) Data represent means ± s.d., n=6-9 (D), n=22-59 (L). Statistical significance was determined using ordinary one-way ANOVA with Tukey's multiple comparisons test; ***P*<0.01, *****P*<0.0001, n.s. = non-significant. The exact number of biological replicates per genotype (n) and *P*-values are specified in Supplementary Dataset 2.

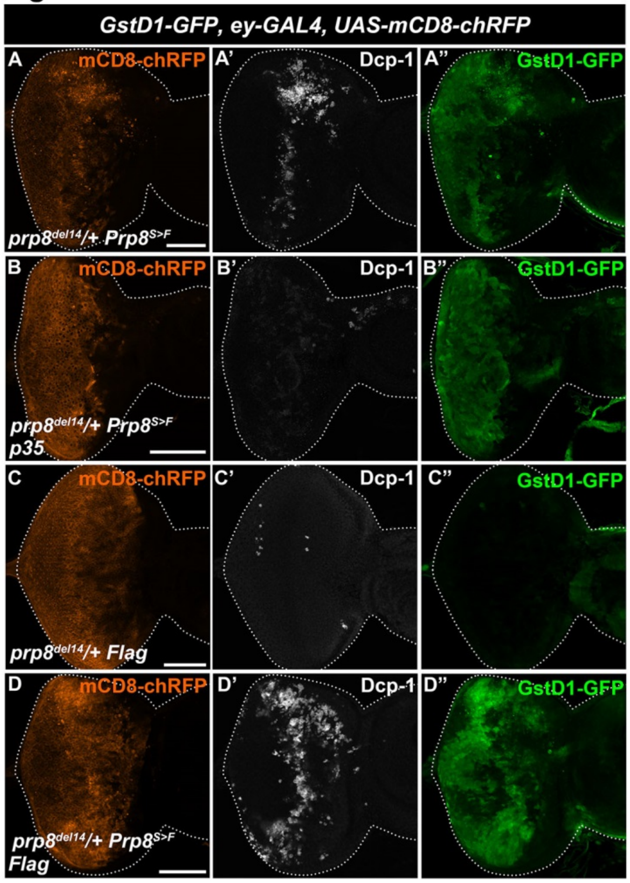

Fig. S6. Blocking apoptosis does not suppress Prp8$^{S>F}$-mediated GstD1 induction.

(**A-D**) Eye-specific expression of Prp8$^{S>F}$ in the *prp8^{del14}*/+ heterozygous background using the *ey-Gal4* driver induces apoptosis (A') and activity of the *GstD1-GFP* reporter (A''). While co-expression of p35 effectively suppresses Prp8$^{S>F}$-induced apoptosis (B') it does not block upregulation of the *GstD1-GFP* reporter (B''). Expression of the mock Flag tripeptide alone does not induce cell death or the *GstD1-GFP* reporter (C). Flag co-expression does not remedy the phenotypic consequences of Prp8$^{S>F}$ (D).

Disease Models & Mechanisms • Supplementary information

Table S1. List of *Drosophila* lines.

Name	Genotype	Source	Identifier
**ey>**	w^{*}; P{$w^{+m^{*}}$=GAL4-ey.H}3-8	Bloomington Drosophila Stock Center	RRID:BDSC_5534
**UAS-mCD8.ChRFP**	w^{*}; P{$w^{+m^{*}}$=UAS-mCD8.ChRFP}2	Bloomington Drosophila Stock Center	RRID:BDSC_27391
**ey>mCD8.ChRFP**	w; P{$w^{+m^{*}}$=GAL4-ey.H}3-8, UAS-mCD8.ChRFP/ CyO	this study	N/A
**GstD1-GFP, ey>mCD8.ChRFP**	w; P{$w^{+m^{*}}$=GAL4-ey.H}3-8, UAS-mCD8.ChRFP, GstD1-GFP/ CyO	this study	N/A
**GstD1-GFP**	w; GstD1-GFP/ CyO	Sykiotis and Bohmann (2008)	N/A
**GstD1-GFP, ey>mCD8.ChRFP, p35**	w; P{$w^{+m^{*}}$=GAL4-ey.H}3-8, UAS-mCD8.ChRFP, UAS-p35, GstD1-GFP/ CyO	this study	N/A
**GstD1-GFP, ey>mCD8.ChRFP, Flag**	w; P{$w^{+m^{*}}$=GAL4-ey.H}3-8, UAS-mCD8.ChRFP, pTFW, GstD1-GFP/ CyO	this study	N/A
**dpp>**	y^{1} w^{67c23}; P{w^{+mC}=dpp-GAL4.PS}6A/ TM3, Ser^{1}	Bloomington Drosophila Stock Center	RRID:BDSC_7007
**dpp>mCD8.ChRFP**	w; ey-Gal4, UAS-mCD8.ChRFP/ CyO	this study	N/A
**phm>**	y^{1} w[*]; P{w^{+mC} =phm-GAL4.O}22	Ono et al. (2006)	RRID:BDSC_80577
**phm>mCD8::GFP**	w;; phm-GAL4, UAS-mCD8::GFP.L/ TM6B	Ono et al. (2006)	N/A
**GMR>**	w^{*}; P{w^{+mC} =GAL4-ninaE.GMR}12	Bloomington Drosophila Stock Center	RRID:BDSC_1104
**UAS-myr-mRFP**	w^{1118}; P{w w^{+mC} =UAS-myr-mRFP}1	Bloomington Drosophila Stock Center	RRID:BDSC_7118
**GMR>myr-mRFP**	w; GMR-Gal4, UAS-myr-mRFP/ CyO	this study	N/A
**ey MARCM>>FRT82B Green**	eyFLP; act>y^{+}>Gal4, UAS-GFP; P{$ry^{+t7.2}$=neoFRT}82B tub-Gal80	Pagliarini and Xu (2003)	N/A
**ey MARCM>>FRT42D Green**	eyFLP; P{$ry^{+t7.2}$=neoFRT }42D tub-Gal80/ T(2;3)B3, CyO:TM6 Tb^{1}/ act>y+>Gal4, UAS-GFP	this study	N/A
**FRT42D**	P{$ry^{+t7.2}$=neoFRT}42D; ry^{605}	Bloomington Drosophila Stock Center	RRID:BDSC_1802

**FRT82B**	*P{ry*$^{+t7.2}$*=neoFRT}82B ry*^{605}	Bloomington Drosophila Stock Center	RRID:BDSC_2035
**Act5C-cas9, Lig4[169]**	*y*^{1}*, M(Act5C-Cas9)ZH-2A, w*^{1118}*, Lig4*^{169}	Zhang et al. (2014)	RRID:BDSC_54590
**sgRNA-Prp8**	*v*^{1}*;; pCFD4-U6:1-U6:3 sgRNA-Prp8/ TM6b*	this study	N/A
**nos-phiC31int;;attP2**	*y*^{1} *sc*^{1} *v*^{1} *P{y*$^{+t7.7}$*=nos-phiC31\int.NLS}X; P{y*$^{+t7.7}$*=CaryP}attP2*	Bloomington Drosophila Stock Center	RRID:BDSC_25710
**Prp8**^{wt}	*w;; pUAST-attB-Prp8*^{wt} attP-9A / *TM6B*	this study	N/A
**Prp8**$^{H>R}$	*w;; pUAST-attB-prp8*^{H2369R} attP-9A/ *TM6B*	this study	N/A
**Prp8**$^{H>P}$	*w;; pUAST-attB-prp8*^{H2369P} attP-9A / *TM6B*	this study	N/A
**Prp8**$^{Y>N}$	*w;; pUAST-attB-prp8*^{Y2395N} attP-9A / *TM6B*	this study	N/A
**Prp8**$^{F>L}$	*w;; pUAST-attB-prp8*^{F2374L} attP-9A / *TM6B*	this study	N/A
**Prp8**$^{R>G}$	*w;; pUAST-attB-prp8*^{H2370G} attP-9A / *TM6B*	this study	N/A
**Prp8**$^{S>F}$	*w;; pUAST-attB-prp8*^{S2178F} attP-9A / *TM6B*	this study	N/A
**Prp8**$^{R>K}$	*w;; pUAST-attB-prp8*^{R2370K} attP-9A / *TM6B*	this study	N/A
**Prp8**$^{R>S}$	*w;; pUAST-attB-prp8*^{R2370S}*/ TM6B*	this study	N/A
**Prp8**$^{P>T}$	*w;; pUAST-attB-prp8*^{P2361T} attP-9A / *TM6B*	this study	N/A
**Flag::Prp8**^{wt}	*w;; pUAST-attB-Flag::Prp8*^{wt} attP2/ *TM6B*	this study	N/A
**Flag::Prp8**$^{H>R}$	*w;; pUAST-attB-Flag::Prp8*^{H2369R} attP2/ *TM6B*	this study	N/A
**Flag::Prp8**$^{H>P}$	*w;; pUAST-attB-Flag::Prp8*^{H2369P} attP2/ *TM3*	this study	N/A
**Flag::Prp8**$^{F>L}$	*w;; pUAST-attB-Flag::Prp8*^{F2374L} attP2/ *TM6B*	this study	N/A
**Flag::Prp8**$^{R>K}$	*w;; pUAST-attB-Flag::Prp8*^{R2370K} attP2/ *TM6B*	this study	N/A
**Flag::Prp8**$^{P>T}$	*w;; pUAST-attB-Flag::Prp8*^{P2361T} attP2/ *TM6B*	this study	N/A
**Flag::Prp8**$^{S>F}$	*w;; pUAST-attB-Flag::Prp8*^{S2178F} attP2/ *TM6B*	this study	N/A
**Flag::Prp8**$^{R>S}$	*w;; pUAST-attB-Flag::Prp8*^{R2370S} attP2/ *TM6B*	this study	N/A
**Flag**	*w; pTFW*	this study	N/A

**FRT42D, prp8^{del14}**	w; P{ry$^{+t7.2}$=neoFRT }42D, prp8^{del14}/ CyO, P{ActGFP}JMR1	this study	N/A
**FRT42D, prp8^{del14}; Prp8$^{S>F}$**	w; P{ry$^{+t7.2}$=neoFRT }42D, prp8^{del14}/ T(2;3)B3, CyO:TM6 Tb^{1}/ pUAST-attB-Prp8^{S2178F} attP-9A	this study	N/A
**FRT42D, prp8^{del14}; Prp8^{wt}**	w; P{ry$^{+t7.2}$=neoFRT}42D, prp8^{del14}/ T(2;3)B3, CyO:TM6 Tb^{1}/ pUAST-attB-Prp8^{wt} attP-9A	this study	N/A
**FRT42D, prp8^{del14}; Prp8$^{F>L}$**	w; P{ry$^{+t7.2}$=neoFRT}42D, prp8^{del14}/ T(2;3)B3, CyO:TM6 Tb^{1}/ pUAST-attB-Prp8^{F2374L} attP-9A	this study	N/A
**FRT42D, prp8^{del14}; Prp8$^{H>R}$**	w; P{ry$^{+t7.2}$=neoFRT}42D, prp8^{del14}/ T(2;3)B3, CyO:TM6 Tb^{1}/ pUAST-attB-Prp8^{H2369R} attP-9A	this study	N/A
**prp8^{RNAi}**	w; UAS-prp8$^{RNAi\ [GD18565]}$	Vienna Drosophila Resource Center	N/A
**prp8^{RNAi}, FRT82B**	w; UAS-prp8$^{RNAi\ [GD18565]}$; P{ry$^{+t7.2}$=neoFRT}82B	this study	N/A
**FRT42D; Prp8^{wt}**	w; P{ry$^{+t7.2}$=neoFRT}42D; pUAST-attB-Prp8^{wt} attP-9A/ TM6B	this study	N/A
**p35**	w; UAS-p35	Bloomington Drosophila Stock Center	RRID: BDSC_5072
**FRT42D prp8^{KG03188}**	w; P{ry$^{+t7.2}$=neoFRT}42D P{y$^{+mDint2}$ w$^{BR.E.BR}$=SUPor-P}Prp8^{KG03188}/ CyO, y^{+}	Kyoto stock Center 111506	
**prp8^{KG03188}**	P{SUPor-P}Prp8 Prp8^{KG03188}/ CyO, P{ActGFP}JMR1	Bloomington Drosophila Stock Center	RRID:BDSC_13006
**FRT42D prp8^{KG03188}; Prp8$^{F>L}$**	w; P{ry$^{+t7.2}$=neoFRT}42D P{y$^{+mDint2}$ w$^{BR.E.BR}$=SUPor-P}Prp8^{KG03188}/ T(2;3)B3, CyO:TM6 Tb^{1}/pUAST-attB-Prp8^{F2374L} attP-9A	Kyoto stock Center 111506	
**FRT42D prp8^{KG03188}; Prp8^{wt}**	w; P{ry$^{+t7.2}$=neoFRT}42D P{y$^{+mDint2}$ w$^{BR.E.BR}$=SUPor-P}Prp8^{KG03188}/ T(2;3)B3, CyO:TM6 Tb^{1}/ pUAST-attB-Prp8^{wt} attP-9A	Bloomington Drosophila Stock Center	RRID:BDSC_13006
**FRT42D, prp8^{KG03188}; Prp8$^{S>F}$**	w; P{ry$^{+t7.2}$=neoFRT}42D, prp8^{del14}/ T(2;3)B3, CyO:TM6 Tb^{1}/ pUAST-attB-Prp8^{S2178F} attP-9A	this study	N/A

Disease Models & Mechanisms • Supplementary information

Table S2. List of vectors and plasmids.

Name	Source	Catalog Number	Identifier
**pDest17**	Thermo Scientific	Cat. #11803012	N/A
**pDest17 Prp8-CTD**	this study		N/A
**pCFD4-U6:1_U6:3tandemgRNAs**	Port et al. (2014), Addgene	Cat. #49411	N/A
**CFD4-U6:1-U6:3 Prp8 sgRNA**	this study		N/A
**pAW-GAL4**	Y. Hiromi		N/A
**pIE-EGFP**			N/A
**pUAST-attB**	Drosophila Genomics Resource Center	Cat. #1419	N/A
**pENTR4 dual selection**	Thermo Scientific	Cat. #A10465	N/A
**pTFW**	Drosophila Genomics Resource Center	Cat. #1115	N/A
**pUAST-attB-Prp8[H2369R]**	this study		N/A
**pUAST-attB-Prp8[H2369P]**	this study		N/A
**pUAST-attB-Prp8[Y2395N]**	this study		N/A
**pUAST-attB-Prp8[F2374L]**	this study		N/A
**pUAST-attB-Prp8[H2370G]**	this study		N/A
**pUAST-attB-Prp8[wt]**	this study		N/A
**pUAST-attB-Prp8[S2178F]**	this study		N/A
**pUAST-attB-Prp8[R2370K]**	this study		N/A
**pUAST-attB-Prp8[R2370S]**	this study		N/A
**pUAST-attB-Prp8[P2361T]**	this study		N/A
**pUAST-attB-Flag::Prp8[H2369R]**	this study		N/A
**pUAST-attB-Flag::Prp8[H2369P]**	this study		N/A
**pUAST-attB Flag::Prp8[wt]**	this study		N/A
**pUAST-attB-Flag::Prp8[R2370K]**	this study		N/A
**pUAST-attB-Flag::Prp8[P2361T]**	this study		N/A
**pUAST-attB-Flag::Prp8[S2178F]**	this study		N/A
**pUAST-attB-Flag::Prp8[R2370S]**	this study		N/A
**pUAST-attB-Flag::Prp8[F2374L]**	this study		N/A
**pENTR4-Prp8[R2370K]**	this study		N/A
**pENTR4-Prp8[R2370S]**	this study		N/A
**pENTR4-Prp8[H2369R]**	this study		N/A

Disease Models & Mechanisms • Supplementary information

**pENTR4-Prp8[S2178F]**	this study		N/A
**pENTR4-Prp8[F2374L]**	this study		N/A
**pENTR4-Prp8[Y2395N]**	this study		N/A
**pENTR4-Prp8[H2370G]**	this study		N/A
**pENTR-Prp8[H2369P]**	this study		N/A
**pENTR4-Prp8[P2361T]**	this study		N/A
**pENTR4-Prp8[wt]**	this study		N/A
**PTFW-Prp8[wt]**	Claudius et al. (2014)		N/A

Table S3. List of oligonucleotides.

Name	Sequence	Purpose
**sgRNA_pCFD4_Prp8_Intron_12_For**	TATATAGGAAAGATATCCGGGTGAACTTCGTACTAGTACATATGCTAAGTGTTTTAGAGCTAGAAATAGCAAG	cloning
**sgRNA_pCFD4_Prp8_3'UTR_1_Rev**	ATTTTAACTTGCTATTTCTAGCTCTAAAACTAAGACTCCATATGCGCACCGACGTTAAATTGAAAATAGGTC	cloning
**Spok iQ For**	GCTCTTTGGCGGTGATCGAAACAA	qPCR
**Spok iQ Rev**	CGCCGAGCTAAATTTCTCCGCTTT	qPCR
**Spok iQ For intron**	CGAGAATTTGGTTGCCAGCGAAACA	qPCR
**Spok iQ Rev intron**	GCCATCCTCTTAAGGAGTGTGGTCAT	qPCR
**RpL49 For**	TCCTACCAGCTTCAAGATGAC	qPCR
**RpL49 Rev**	CACGTTGTGCACCAGGAACT	qPCR
**Prp8_ H2369R_For**	ATCACGAGTTGCGTCGCACCTCGCA	mutagenesis
**Prp8_ H2369R_Rev**	TGCGAGGTGCGACGCAACTCGTGAT	mutagenesis
**Prp8_H2309P_For**	ATCACGAGTTGCCTCGCACCTC	mutagenesis
**Prp8_H2309P_Rev**	GAGGTGCGAGGCAACTCGTGAT	mutagenesis
**Prp8_Y2334N_for**	CGGGAGGATGTGAACGCGTAAGCGC	mutagenesis
**Prp8_Y2334N_rev**	GCGCTTACGCGTTCACATCCTCCCG	mutagenesis
**Prp8_F2374L_For**	ACCTCGCATTTACTGCTCTTCTCG	mutagenesis
**Prp8_F2374L_Rev**	CGAGAAGAGCAGTAAATGCGAGGT	mutagenesis
**Prp8_R2310G_For**	CACGAGTTGCATGGCACCTCGCATT	mutagenesis
**Prp8_R2310G_Rev**	AATGCGAGGTGCCATGCAACTCGTG	mutagenesis
**Prp8_S2118F_For**	GTCACGATCTTTGATCTGCGGGCC	mutagenesis
**Prp8_S2118F_Rev**	GGCCCGCAGATCAAAGATCGTGAC	mutagenesis
**Prp8_R2310K_For**	ACGAGTTGCATAAGACCTCGCATTT	mutagenesis
**Prp8_R2310K_Rev**	AAATGCGAGGTCTTATGCAACTCGT	mutagenesis
**Prp8_R2310S_For**	ACGAGTTGCATTCCACCTCGCATTT	mutagenesis
**Prp8_R2310S_Rev**	AAATGCGAGGTGGAATGCAACTCGT	mutagenesis
**Prp8_P2361T_For**	AGCTGGCCAACACGAAGGAGTTCTA	mutagenesis
**Prp8_P2361T_Rev**	TAGAACTCCTTCGTGTTGGCCAGCT	mutagenesis
**PRP8 cDNA Bcl1 Forward**	AAATGATCACGATGTCCATTCCGCCGTACATG	cloning
**PRP8 cDNA Not1 Rev**	AAAGCGGCCGCGCTTACGCGTACACATCCTCCC	cloning
**PRP8 CTD EcoRI For**	AAAGAATTCCCATGCAGACCAAGGAGCAAAACCAAC	cloning
**ets21c iQ For**	ATTAATGCCATGCATCAGGATGTCCG	qPCR
**ets21c iQ Rev**	GTGGGAACTTCCGTCTCCTTCG	qPCR
**GstE6 iQ For**	CCAAGGAGCGATACGATGCCA	qPCR
**GstE6 iQ Rev**	CCACGAAGGCCTCAAGGGAG	qPCR

Disease Models & Mechanisms • Supplementary information

**dilp8 iQ For**	GCACAACAAGCATCACTACATCA	qPCR
**dilp8 iQ Rev**	GTTGTAGGACCTGCTCGAGTG	qPCR
**arc1 iQ For**	GCTGAACATCAAGTACCGCAAGCA	qPCR
**arc1 iQ Rev**	GTGTTCTTTGCTGTGGCAAGCTGT	qPCR
**CG42260 iQ For**	TACTCAATATGGGCACCGC	qPCR
**CG42260 iQ Rev**	CTTCAGCACGTCCCACATG	qPCR

Supplementary Dataset 1

Click here to download Supplementary Dataset 1

Supplementary Dataset 2

Click here to download Supplementary Dataset 2
